# Strategies to improve the implementation of preventive care in primary care: a systematic review and meta-analysis

**DOI:** 10.1186/s12916-024-03588-5

**Published:** 2024-09-27

**Authors:** Laura Heath, Richard Stevens, Brian D. Nicholson, Joseph Wherton, Min Gao, Caitriona Callan, Simona Haasova, Paul Aveyard

**Affiliations:** 1 Nuffield Department of Primary Care Health Sciences, University of Oxford, Radcliffe Primary Care Building, Radcliffe Observatory Quarter, Woodstock Road, Oxford, OX2 6GG UK; 2 Department of Marketing, University of Lausanne, Quartier UNIL-Chamberonne, Lausanne, Quartier CH-1015 Switzerland

**Keywords:** Prevention, Primary care, Implementation, Smoking, Obesity, Physical activity, Exercise, Alcohol

## Abstract

**Background:**

Action on smoking, obesity, excess alcohol, and physical inactivity in primary care is effective and cost-effective, but implementation is low. The aim was to examine the effectiveness of strategies to increase the implementation of preventive healthcare in primary care.

**Methods:**

CINAHL, CENTRAL, The Cochrane Database of Systematic Reviews, Dissertations & Theses – Global, Embase, Europe PMC, MEDLINE and PsycINFO were searched from inception through 5 October 2023 with no date of publication or language limits. Randomised trials, non-randomised trials, controlled before-after studies and interrupted time series studies comparing implementation strategies (team changes; changes to the electronic patient registry; facilitated relay of information; continuous quality improvement; clinician education; clinical reminders; financial incentives or multicomponent interventions) to usual care were included. Two reviewers screened studies, extracted data, and assessed bias with an adapted Cochrane risk of bias tool for Effective Practice and Organisation of Care reviews. Meta-analysis was conducted with random-effects models. Narrative synthesis was conducted where meta-analysis was not possible. Outcome measures included process and behavioural outcomes at the closest point to 12 months for each implementation strategy.

**Results:**

Eighty-five studies were included comprising of 4,210,946 participants from 3713 clusters in 71 cluster trials, 6748 participants in 5 randomised trials, 5,966,552 participants in 8 interrupted time series, and 176,061 participants in 1 controlled before after study. There was evidence that clinical reminders (OR 3.46; 95% CI 1.72–6.96; *I*^2^ = 89.4%), clinician education (OR 1.89; 95% CI 1.46–2.46; *I*^2^ = 80.6%), facilitated relay of information (OR 1.95, 95% CI 1.10–3.46, *I*^2^ = 88.2%), and multicomponent interventions (OR 3.10; 95% CI 1.60–5.99, *I*^2^ = 96.1%) increased processes of care. Multicomponent intervention results were robust to sensitivity analysis. There was no evidence that other implementation strategies affected processes of care or that any of the implementation strategies improved behavioural outcomes. No studies reported on interventions specifically designed for remote consultations. Limitations included high statistical heterogeneity and many studies did not account for clustering.

**Conclusions:**

Multicomponent interventions may be the most effective implementation strategy. There was no evidence that implementation interventions improved behavioural outcomes.

**Trial registration:**

PROSPERO CRD42022350912.

**Supplementary Information:**

The online version contains supplementary material available at 10.1186/s12916-024-03588-5.

## Background

Smoking, obesity, alcohol intake, and physical inactivity bring forward the onset of chronic disease, multimorbidity, and premature death. Compared to individuals with no behavioural risk factors, those with 2 or more risk factors (smoking, obesity, alcohol intake and physical inactivity) can expect to live on average 12 years fewer [1]. In 2015, 30.3% and 15.5% of global disease burden were accounted for by behavioural factors and metabolic factors, respectively [2]. Differences in the prevalence of these risk factors explain a substantial portion of the health gap between those from affluent and deprived areas [3, 4]. One way health systems address this is through preventive healthcare. This includes supporting behaviour change via brief opportunistic interventions and referral to further support [5, 6]. Systematic reviews of randomised trials and modelling from their results show that opportunistic screening for and intervention to support behaviour change is effective and cost-saving for smoking cessation [7, 8]; effective and cost-effective for reducing hazardous drinking [9, 10]; effective and cost-effective for weight loss in obesity [11, 12]; effective and may be cost-effective for physical inactivity [5, 13].

These behaviour changes reduce the development of type 2 diabetes, cardiovascular disease, cancer and premature mortality [14]. They have been shown to be feasible in primary care and equitable in their impact [15]. Increasing preventive care delivery can also reduce the environmental impact of healthcare and support the transition to more sustainable healthcare systems [16]. Optimising the implementation of these evidence-based interventions is a health system priority [17]. However, the rate of intervention by primary healthcare professionals, who are well-placed to deliver them, is low [18–20]. For example, in the UK in 2020, the rate of advice for weight management (8 events per hundred patients per year), physical inactivity (4 events per hundred patients per year) and excessive alcohol intake (4 events per hundred patients per year) was low compared to the prevalence of overweight or obesity (approximately 60%), of physical inactivity (approximately 30%) and of harmful alcohol consumption (approximately 20%) [21–23]. Given their effectiveness and cost-effectiveness, governments and health systems have attempted to increase the implementation of this type of preventive healthcare [17, 24]. We therefore conducted a systematic review and meta-analysis to examine the effectiveness of different implementation strategies (Table 1) compared to usual care, in adults in a primary healthcare setting to increase both process and behavioural outcomes for smoking, obesity, excessive alcohol consumption and physical inactivity.

**Table 1:** Taxonomy for implementation strategies

*Implementation strategies targeting the healthcare system.*
1. *Preventive care management.* The implementation of a system for organising preventive care, with a person/organisation responsible for monitoring delivery or performance.
2. *Team changes.* An additional team member/s or role added with responsibility to increase the delivery of preventive care.
3. *Electronic patient record.* Beyond collection of routine data, the electronic health record must be used specifically as an intervention to target individuals and to enable the delivery of preventive care.
4. *Facilitated relay of information to clinicians.* Information collected, separately to the main clinical record which is relayed to clinicians to facilitate preventive care.
5. *Continuous quality improvement (QI).* Systems level QI is an iterative process, with defined methods for identifying inefficiencies in preventive care system wide, with a plan for action and recollection of data on performance.
*Implementation strategies targeting healthcare professionals.*
6. *Audit and feedback.* A summary of the preventive care delivered by a clinic or healthcare professional over time, which is then relayed back to the clinic or individual for reflection.
7. *Clinician education.* An educational programme for preventive care delivered to all fully qualified healthcare professionals in primary care.
8. *Clinical reminders.* In-consultation reminders to deliver any aspect of preventive care.
9. *Financial incentives.* Pay for performance schemes where payment was given to deliver a specific aspect of preventive care.
*Other*
10. *Multi-component interventions.* Interventions that combined two or more of the above strategies.

The way primary health care is being delivered is also changing as more consultations are being delivered remotely (via telephone, video, email or text message), and this may affect implementation efforts [25, 26]. Therefore, we also aimed to examine the effectiveness of implementation strategies in this current context.

## Methods

A full protocol was prospectively published on the International Prospective Register of Systematic Reviews (PROSPERO) [27]. This followed the Cochrane Effective Practice and Organisation of Care (EPOC) guidelines for an implementation systematic review and is reported according to the preferred reporting items for systematic reviews and meta-analysis (PRISMA) statement [28, 29].

### Data sources and searches

We searched the Cumulated Index in Nursing and Allied Health Literature (CINAHL), The Cochrane Central Register of Controlled Trials (CENTRAL), The Cochrane Database of Systematic Reviews, Dissertations & Theses – Global, Excerpta Medica Database (Embase), Europe PubMed Central (PMC), Medical Literature Analysis and Retrieval System Online (MEDLINE) and PsycINFO for studies until 5th October 2023. The references of included studies were manually searched for studies missed in the database search. The complete database search strategy is included in Additional file 1.

### Study selection

The population considered for inclusion were adult patients seeking primary health care, where interventions for behaviour change happen opportunistically. If adolescents were also included in the study population, we only analysed the participants over the age of 18. If it was not possible to separate those under 18 from adult participants, we only included the study if the average age of participants was over 18 years. In this review, we use the National Health Service (NHS) England definition of primary care, including the general practice multidisciplinary team, community pharmacy, dental and optometry services [30]. When it was unclear whether a study was conducted in primary care, a decision was made in consultation with the wider research team which included three primary care physicians, considering whether this was the first point of contact for the patient in the healthcare system. We included cluster randomised trials (cRT), cluster non-randomised trials (cNRT), randomised trials (RT), controlled before-after studies (CBA) and interrupted time series (ITS) studies.

Exclusion criteria were adults seeking care for established disease, e.g. weight loss as a treatment for type 2 diabetes, and people who were receiving palliative care. There were no date or language restrictions.

Interventions included were those both at the health system and health professional level and these were compared to usual care. We included interventions that used one of ten implementation strategies (Table 1) to encourage action on smoking, poor diet, alcohol consumption or physical inactivity. This taxonomy of implementation strategies was adapted from the taxonomy used by the Cochrane EPOC group [31, 32]. A similar approach has been used in another study looking at implementation strategies to optimise care for type 2 diabetes [33].

After deduplication, two reviewers independently screened title and abstracts and then full-text records against prespecified eligibility criteria using a decision flowchart. Covidence screening software (Veritas Health Innovation) was used for deduplication and title, abstract, and full-text screening [34].

### Data extraction and quality assessment

Data were then extracted independently by two reviewers using a piloted Microsoft Excel spreadsheet. Extracted information included baseline characteristics, study design, intervention characteristics and outcome measures. Risk of bias was independently assessed by two authors using the Cochrane EPOC risk of bias tool for the appropriate study design. Disagreements were resolved by the wider team for review.

### Data synthesis and analysis

In line with Donabedian’s three-component approach for measuring the quality of care, the main outcomes were measures that record changes in process (e.g. referral to further support) and behavioural outcome (e.g. smoking cessation or weight loss) of preventive care [35]. Outcomes were grouped by predominant mode of intervention e.g., clinician education or team changes. Outcomes were extracted at 1 year, or the closest measurement to this. Secondary outcomes included patient acceptability and satisfaction with the intervention; healthcare professional acceptability and satisfaction with the intervention; resource use; equity impact, and adverse effects.

Where more than one health behaviour was reported we prioritised a summary statistic of the effect of the intervention on combined health behaviours, the primary outcome of the study, or if neither of these were present, the first reported health behaviour. If more than one process outcome was reported, we took the most distal outcome, e.g. if advice given and referral made were both reported, we used the referral data. We included studies that reported changes in health behaviours (e.g. diet) and outcomes of health behaviours (e.g. weight) and grouped these together for the behavioural outcomes meta-analysis.

We extracted continuous and dichotomous outcomes, converting these where required from a standardised effect measure to an odds ratio (OR) using an adapted Chinn’s method [36]. Where a study did not correct for clustering, we adjusted the width of the confidence interval as described in the Cochrane handbook [37]. The upper quartile intracluster correlation coefficient (ICC) was selected from the included studies. This conservative approach also reduces the influence of outlier values. When the number of cases in the intervention or control group was zero, the Peto method was used to calculate an OR. Calculation details can be found in Additional file 2.

Where an adjusted hazard ratio, incident rate ratio or risk ratio was given by the study, this was taken as a conservative estimate of the odds ratio. If two or more intervention groups existed, we selected the intervention group that most closely represented the implementation strategy of interest. For example, we used the training workshop and usual care arms, and not the free patient education material arm in the Kottke et al. study [38]. Where there were intervention arms of different intensities, these were combined into a single intervention arm. Some studies provided insufficient information to calculate a standardised effect measure. In these cases, the authors were contacted and if no further information was provided, these results were synthesised narratively.

We used random effects meta-analysis to pool study outcomes, given that the true effect of preventive interventions is likely to differ across contexts and health behaviours. Sensitivity analysis (Additional file 3: Fig. S1–S2) used two further models to pool the study outcomes. Firstly, the Hartung-Knapp-Sidik-Jonkman (HKSJ) model variance correction was applied to the standard DerSimonian-Laird model; optionally without truncation of correction factor at 1, to reduce the risk of poor coverage of 95% confidence intervals (CIs). Secondly, the inverse variance heterogeneity (IVhet) model, for meta-analysis of heterogenous studies [39, 40]. Meta-analysis was conducted in Stata, version 14.2 (StataCorp) using the admetan command. The full Stata code is available in Additional file 4. Forest plots were used to display the results of meta-analysis. The *I*^2^ statistic and 95% prediction intervals were calculated to assess heterogeneity.

Additional sensitivity analyses were conducted where possible, excluding firstly studies at high risk of bias, secondly studies where data had to be imputed (Additional file 3: Fig. S3–S4), and lastly when outcomes of health behaviours were reported (e.g. weight) rather than the health behaviour directly (e.g. diet) (Additional file 3: Fig. S5). Subgroup analysis considered each health behaviour separately (Additional file 3: Fig. S6–S7). These analyses were completed where there was more than one study able to be meta-analysed in each subgroup.

### Role of the funding source

This review was funded by the Wellcome Trust who had no role in the design of the study or analysis or interpretation of the data.

## Results

**Fig. 1:**
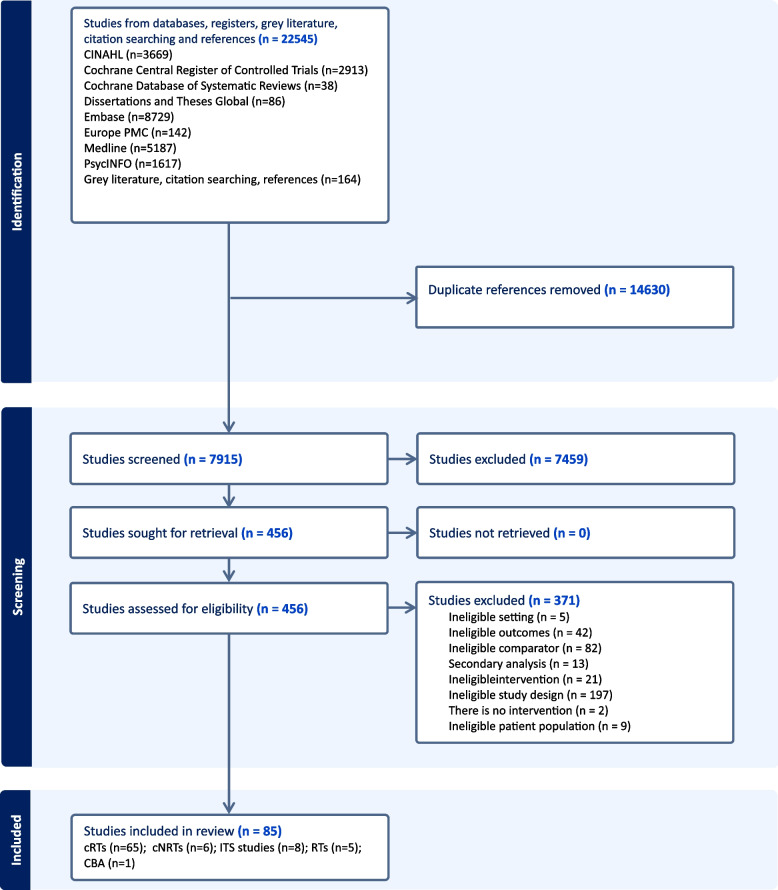
PRISMA flow diagram

Figure 1 shows the flow through the study. The search identified 22,545 unique study titles. After screening, 456 full texts were assessed for eligibility and 85 studies were included.

### Study characteristics

Of the 85 included studies (Additional file 4: Table S1), [38, 41–134] 65 were cRTs, 6 were cNRTs, 8 were ITS studies, 5 were RTs and 1 was a CBA study. The 71 cluster trials had a total of 3713 clusters and 4,210,946 participants. The randomised trials had a total of 6748 participants, the interrupted time series studies used data from 5,966,552 participants 176,061 participants in 1 CBA study. Forty-two studies focussed on smoking, 19 on alcohol, 7 on obesity (poor diet), 2 on physical activity and 15 on multiple health behaviours. The average age of patients and healthcare professionals was 49 and 43 years, respectively. Forty-eight per cent of patients and 45% of healthcare professionals were male. Of the small number of studies that reported ethnicity, 67% of patients and 69% of healthcare professionals were White. The most common countries were the USA (*n* = 42), Europe (*n* = 15) and the UK (*n* = 13). The mean (standard deviation) follow-up time for ITS studies was 44 (42) months and for the other study types was 10 (6) months.

No studies examined the impact of preventive care management interventions or audit and feedback interventions. Three studies investigated the impact of team changes; 5 studies investigated the electronic patient registry; 5 studies investigated the facilitated relay of information; 1 study investigated continuous quality improvement; 37 studies investigated clinician education interventions; 9 investigated clinical reminders; 7 investigated financial incentives and 18 investigated multi-component interventions. Of the studies, 68 were included in meta-analyses and 17 studies were synthesised narratively. No studies examined implementation strategies specifically for remote consultations in primary care.

### Risk of bias

Risk of bias was assessed to be low in 25 studies, unclear in 24 studies and high in 36 studies (Tables 2 and 3). Removing studies at high risk of bias in the sensitivity analysis did not significantly change the meta-analysis results (Additional file 3: Fig S3–S4).

**Table 2:**
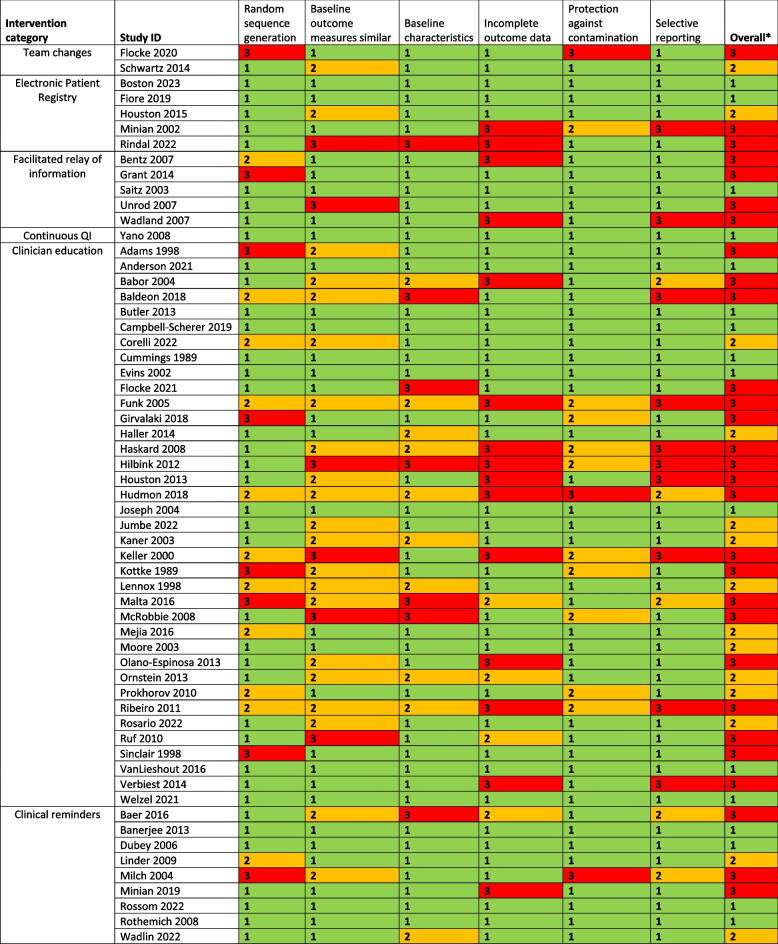

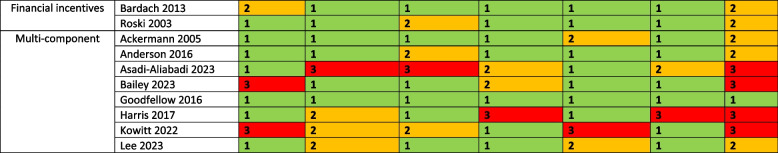
Risk of bias assessment for cluster-randomised, cluster non-randomised, and controlled pre-post studies

**Table 3:**
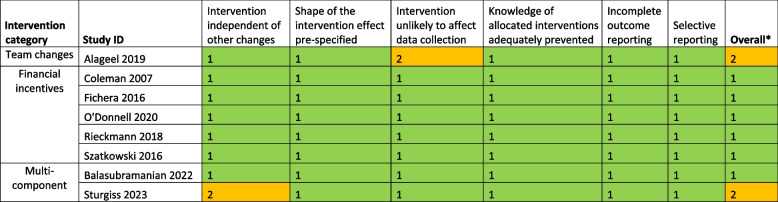
Risk of bias assessment for interrupted time series (ITS) studies

1 = low risk of bias; 2 = unclear risk of bias; 3 = high risk of bias

*Overall score is the highest risk of bias score assigned to the study

### Process outcomes

**Fig. 2:**
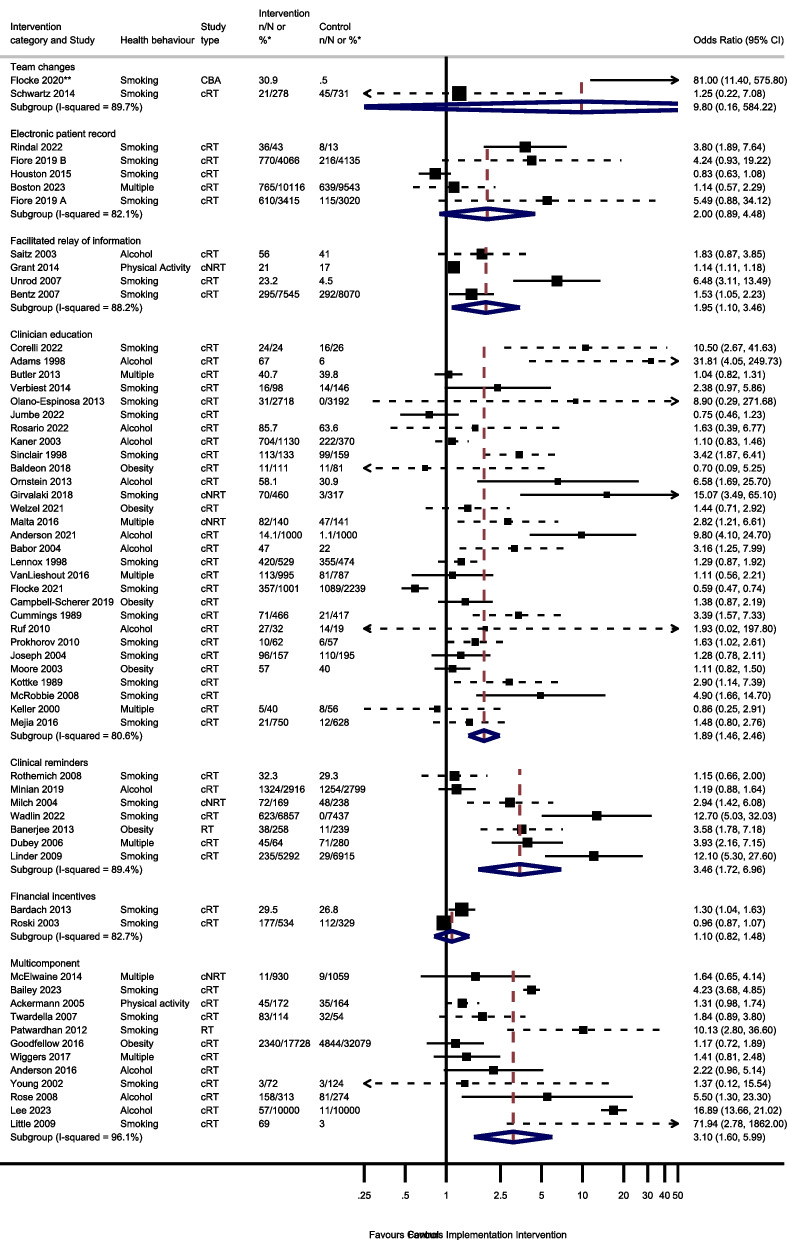
Odds of improving process outcomes between implementation interventions and control groups using random effects meta-analysis *n/N or % given where available in the paper. ** Flocke study estimate not visible for reasons of scale. Note: Dashed line indicates studies for which approximate data had to be used

Sixty studies were included in the process outcome meta-analysis from 7 intervention groups: 7 clinical reminder studies; 29 clinician education studies, 4 electronic patient registry studies, 4 facilitated relay of information studies, 2 financial incentive studies, 12 multi-component studies and 2 team change studies (Fig. 2).

Three studies reported the effect of team changes on process outcomes. One ITS study was not included in the meta-analysis. This study found team changes resulted in a significant increase in the number of people receiving an appropriate weight management referral or smoking cessation intervention [44]. The meta-analysis of the remaining 2 studies reported imprecise results (OR 9.80, 95% CI 0.16–584.22, *I*^2^ = 89.7%, 95% CI 62–97%).

Four studies examined the effect of changing the electronic patient record. One of the 4 electronic patient record studies had 2 separate analyses for 2 different healthcare systems [66]. There was no evidence that amending the electronic patient record increased preventive processes (OR 2.00; 95% CI 0.89–4.48, *I*^2^ = 82.1%, 95% CI 59–92%).

Five studies reported the effect of the facilitated relay of information on preventive care, with 4 included in the meta-analysis. One study was unable to complete clinician and practice level analyses because the sample was too small [128]. There was evidence that facilitated relay of information interventions significantly increased preventive processes (OR 1.95, 95% CI 1.10–3.46, *I*^2^ = 88.2%, 95% CI 72–95%).

Thirty-one studies examined the effects on process outcomes of clinician education interventions. Two studies were unable to be included in the analysis [69, 80]. One study showed no evidence of a significant difference between intervention and control groups being signposted to quitline services by pharmacy staff [80]. The second study found that training and support for GPs significantly increased the rate of implementation of brief interventions for alcohol [69]. Meta-analysis of the remaining 29 studies showed a significant increase in preventive process outcomes (OR 1.89, 95% CI 1.46–2.46, *I*^2^ = 80.6%, 95% CI 73–86%).

Seven of the 8 clinical reminder studies with process outcomes were able to be meta-analysed. These showed a statistically significant increase in health process outcomes (OR 3.46, 95% CI 1.72–6.96, *I*^2^ = 89.4%, 95% CI 81–94%). One study not included in the meta-analysis reported that there were no significant differences between groups in changes in the percentages of patients who had a nutrition counselling visit when reminders and alerts were added to the records of patients with overweight or obesity [135].

Six studies reported the effect of financial incentives on process outcomes. There were differences in the way that results were reported for four ITS studies, precluding meta-analysis. Three reported statistically significant positive associations between financial incentives and greater alcohol and smoking advice/interventions, [100, 122, 136] and one reported a positive association between the financial incentive and clinicians’ alcohol advice or intervention without commenting on statistical significance [106]. Two cRTs reported process outcomes [55, 111]. There was no evidence from the meta-analysis of these studies that financial incentives increased the delivery of preventive processes (OR 1.10, 95% CI 0.82–1.48, *I*^2^ = 82.7%, 95% CI 27–96%).

Seventeen multicomponent intervention studies reported process outcomes, of which 12 were included in the meta-analysis. Two ITS studies not included in the meta-analysis found that multicomponent interventions were associated with an increase in the number of people screened for tobacco use or receiving a smoking cessation intervention, [52] and an increase in alcohol recording [125]. One study not included in the meta-analysis found a statistical increase in the percentage of tobacco users who received a cessation intervention [85]. However, another study not included in the meta-analysis reported no evidence that patients were more likely to receive behavioural advice or referral at follow-up for any health behaviour, [74] and another found no evidence for a change in alcohol screening after implementing a multicomponent intervention [125]. Of the 12 studies included in the meta-analysis, there was evidence that multicomponent interventions increased the process outcomes (OR 3.10, 95% CI 1.60–5.99, *I*^2^ = 96.1%, 95% CI 95–97%).

### Sensitivity and subgroup analysis

Sensitivity analysis showed that multicomponent interventions were the only category of intervention that showed evidence of increased process outcomes in all 3 meta-analysis models (random effects, HKSJ, and IVhet) (Additional file 3: Fig. S1). Removing studies at high risk of bias or studies that required imputed data did not significantly change the results (Additional file 3: Fig. S3). Subgroup analysis showed that there was no evidence that clinician education or multicomponent interventions that targeted multiple health behaviours increased process outcomes (Additional file 3: Fig. S6).

### Behavioural outcomes

**Fig. 3:**
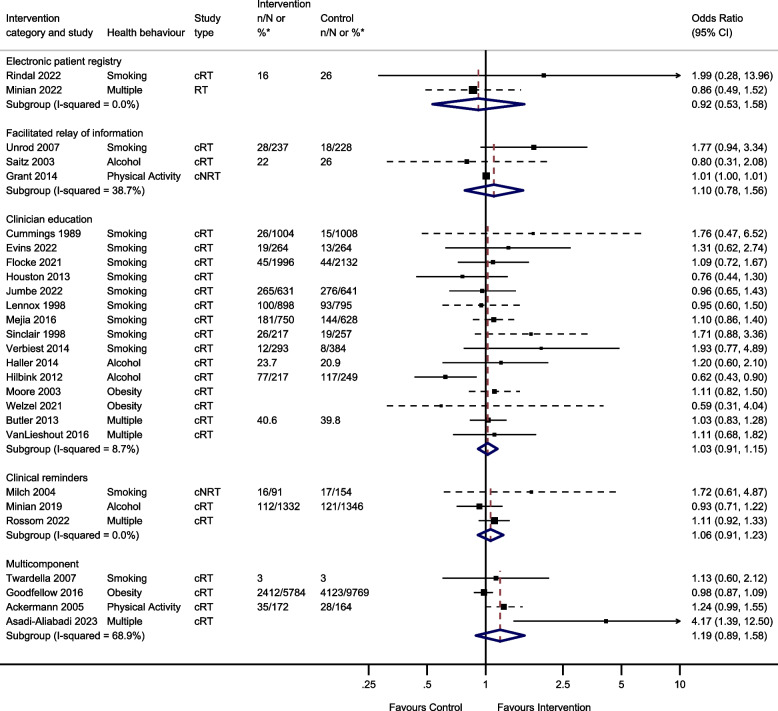
Odds of improving behavioural outcomes between implementation interventions and control groups using random effects meta-analysis *n/N or % given where available in the paper. Note: Dashed line indicates studies for which approximate data had to be used

Thirty-nine studies reported behavioural outcomes and were able to be meta-analysed, assessing the effect of 5 intervention modes. This included 15 studies of clinician education; 3 studies of clinical reminders; 2 electronic patient registry studies; 3 facilitated relay of information studies, and 4 studies of multicomponent interventions (Fig. 3).

Two studies reported the effect of team changes on behavioural outcomes. These were unable to be meta-analysed due to differences in the reporting of data. One found that individuals who were in the intervention group were significantly more likely than controls to have a lower body mass index (BMI) and to have quit smoking at the end of the follow-up period, [44] the other found no evidence of a difference in self-reported smoking cessation at 8 months [117].

Two studies investigated the effect of changes to the electronic patient record on behavioural outcomes and were meta-analysed. There was no evidence that changes to the electronic patient registry improved behavioural outcomes (OR 0.92, 95% CI 0.53–1.58, *I*^2^ = 0.0%, 95% CI 0–100%).

Four studies reported the effect of facilitated relay of information on beneficial behavioural outcomes. One study was unable to complete clinician and practice level analyses because the sample was too small [128]. Three studies included in the meta-analysis found no evidence that facilitated relay of information improved behavioural outcomes (OR 1.10, 95% CI 0.78–1.56, *I*^2^ = 38.7%, 95% CI 0–81%).

One study investigated the effect of continuous quality improvement on smoking outcomes [133]. There was no evidence of a difference in smoking cessation between the intervention and the control groups (OR 0.90, 95% CI 0.58–1.39).

Twenty studies reported the effect of clinician education on behavioural outcomes. Five were unable to be included in the meta-analysis due to insufficient information. One study showed a significant increase in smoking cessation in the intervention group compared to controls at 1 year [101]. Another study found the intervention group had a greater reduction in alcohol dependence score during follow-up compared to the control group [105]. However, three studies found no evidence of a difference in rates of smoking cessation, alcohol dependence score or difference in BMI/ weight between intervention and control groups during follow-up [38, 53, 115]. The meta-analysis showed there was no evidence that clinician education (OR 1.03, 95% CI 0.91–1.15, *I*^2^ = 8.7%, 95% CI 0–46%) improved behavioural outcomes.

Four studies reported the effect of clinical reminders on beneficial behavioural outcomes. One study not included in the meta-analysis due to insufficient data showed no evidence of a difference in weight loss between intervention and control groups [50]. The remaining 3 studies included in the meta-analysis showed no evidence that clinical reminders improved behavioural outcomes (OR 1.06, 95% CI 0.91–1.23, *I*^2^ = 0.0%, 95% CI 0–11%).

Two studies reported the effect of financial incentives on behavioural outcomes. One was an ITS study, and the other was a cRT. Due to the difference in reporting of data, these were unable to be meta-analysed. One reported that financial incentives were significantly associated with reduced smoking, but not with reduced BMI or alcohol consumption in the 6 years following the introduction of the financial incentive [65]. The other showed there was no evidence of an effect of financial incentives on smoking cessation at 6 months [111].

Four studies reported the effect of multicomponent interventions. There was no evidence that multicomponent interventions improved behavioural outcomes (OR 1.19, 95% CI 0.89–1.58, *I*^2^ = 68.9%, 95% CI 10–89%).

### Sensitivity and subgroup analysis

Sensitivity analysis showed no significant difference in meta-analysis results using different models (random effects, HKSJ, and IVhet) (Additional file 3: Fig. S2), when excluding studies at high risk of bias, or studies that used imputed data (Additional file 3: Fig. S4). Due to the small number of studies in the meta-analysis, subgroup analysis was only conducted for clinician education studies. There was no evidence that the effect of clinician education varied between smoking, alcohol, obesity, or multiple behavioural outcomes (Additional file 3: Fig. S7). Removing the three studies that measured the outcome of a health behaviour (weight) rather than the health behaviour directly (diet) did not change the result of the meta-analysis (Additional file 3: Fig. S5) [71, 98, 130].

### Secondary outcomes

Most studies did not report data on our secondary outcomes. Three studies reported no adverse effects of the intervention [41, 64, 82]. Thirteen studies reported that training received was useful, relevant, or increased healthcare professional confidence and self-esteem [61, 70, 71, 75, 82, 90, 104, 105, 108, 120, 127, 130, 134]. One electronic health record study commented that eReferral had good reach amongst people without health insurance, and this could help reduce health inequalities [66]. However, another study described how people from deprived communities and smokers were less likely to take up the offer of an additional health check [44].

One study reported on barriers to weight management in follow-up interviews. Healthcare professionals described how too many clinical reminders were counterproductive [50]. A clinical education smoking study in community pharmacies described time constraints, privacy, and part-time staff as barriers to maintaining an accurate clinical record [118]. Healthcare professionals in another clinical education study questioned whether in-depth training for weight management was feasible against other competing clinical demands [98].

Three studies presented data relevant to the cost-effectiveness of the intervention. One calculated the additional cost of clinician-specific feedback at US$65 per estimated quit [128]. Another clinical education study targeting smoking cessation, found the intervention incremental cost per life year gained after 6 months was €969 [101]. Finally, a clinical education study for excess alcohol found a similar cost-effectiveness ratio between control and intervention practices [83].

## Discussion

### Summary

There was some evidence that many implementation strategies including clinical reminders, clinician education, facilitated relay of information and multicomponent interventions increased the occurrence of preventive processes of care. Multicomponent interventions were the most robust in sensitivity analysis. There was some evidence in subgroup analysis that implementation strategies that target multiple behaviours may be less effective than those that target single behaviours. However, there was no consistent evidence that these process changes translated into improved behavioural outcomes.

### Strengths and limitations

Our search strategy was thorough, including 8 databases, grey literature, references, and citation searching, with no date or language constraints. The resulting sample is large with data from over 10 million participants. Included studies came from North America, Europe, South America, the Middle East, and Australia so our results are relevant to many healthcare systems.

Dividing interventions into implementation categories allows the results to be relevant to policy makers and public health professionals, although for a few studies, the predominant implementation strategy was not clear. We resolved these through consensus of all investigators. We followed PRISMA guidance throughout this study (Additional file 6 and Additional file 7).

Many of our studies had a high risk of bias. This was partly due to our decision to include a greater range of study designs and include non-randomised studies. This decision was made as some interventions e.g., financial incentives implemented across an entire health care system cannot practically be randomised in a traditional trial setting. There was also high heterogeneity, especially in the primary analyses. This is not unexpected as, although interventions were of a similar type, they differed in intensity, duration, delivery, and format. In addition, studies were conducted in different countries, healthcare systems with different methods of usual care, and amongst different population demographics, which also likely contributed to the high heterogeneity. In the presence of such high heterogeneity, we considered it useful to conduct meta-analysis and examine statistical significance, but the individual point estimates should not be over-interpreted. Random-effects meta-analysis allows for differences in the intervention effect between studies and provides an estimate of the average intervention effect [137]. We also calculated prediction intervals for each meta-analysis conducted (Additional file 8: Table S1). These confirm the high levels of heterogeneity in the analysis, with a range of ORs plausible for different settings or populations. We did not formally assess for publication bias or other small study effects. We think it unlikely that unreported studies or results would change our conclusions. Only two studies reported information about how the intervention effectiveness differed between socioeconomic groups. Future studies should collect this information to understand which population groups may benefit most.

We had to impute several calculations as many studies had not accounted for clustering, or we had to calculate an OR from available data. Whilst these established methods, they introduce another opportunity for error. Resulting CIs were often wide, reflecting the uncertainty in data from single trials.

When analysing behavioural outcomes, we combined studies that targeted both behaviours directly and those that targeted outcomes of behaviours. One study measured alcohol dependence rather than consumption using the Alcohol Use Disorders Identification Test (AUDIT) score [76]. However, this scoring system is strongly correlated with alcohol consumption, and so we retained this study in the meta-analysis [138, 139]. Another study used a combined metric of smoking status, alcohol consumption, physical activity and diet [58]. We also retained this study in the meta-analysis as most of the measures used (smoking status, alcohol consumption and physical activity) were used by the other studies. It seems unlikely that including these studies would change the conclusion that there was no evidence of effect. Furthermore, a post hoc sensitivity analysis that excluded three studies that measured the outcome of a health behaviour (weight) rather than the health behaviour (diet) directly, did not change the result of the meta-analysis that there was no evidence of effect [71, 98, 130].

The observed difference in process and behavioural outcomes may be because outcomes can be measured more precisely in proximal (process) outcomes, behavioural outcomes take time to emerge, and many studies did not have sufficient follow-up periods to capture changes in patient behaviour. Furthermore, process outcomes are recorded at the time of consultation, while changes in behaviour may have occurred without the patient returning and/or the clinician asking about behaviour and recording the change at a subsequent consultation. Moreover, increases in the recording of processes of care may reflect that clinicians start recording activity that was previously unrecorded. If so, increases in processes of care may occur while changes in patient behaviour would not be expected to increase. This is particularly likely to occur where the intervention is a financial incentive. However, trial data has shown that genuine increases in process outcomes does translate into beneficial behavioural outcomes [11].

### Comparison with existing literature

To our knowledge, this is the first systematic review to investigate implementation strategies for preventive healthcare. Other studies have focussed on quality improvement strategies in chronic disease management or fall prevention. For example, systematic reviews investigating quality improvement strategies in diabetes care found that multicomponent QI programmes may achieve meaningful population improvements for most immediate diabetes outcomes and that interventions at the health system or patient level may be more effective than interventions at the health professional level [33, 140]. A comprehensive systematic review looking at falls prevention found evidence that team changes and multicomponent interventions may reduce falls [141]. Another review reported that clinician education and patient reminders and education were the most effective strategies for reducing systolic and diastolic blood pressure respectively [142]. Our review supports these findings for the implementation of preventive healthcare in primary care. This suggests that quality improvement strategies may be generalisable across clinical targets. However, due to the high heterogeneity observed in this review, the results should be interpreted cautiously. Future research should collect data about which population subgroups may benefit most from these interventions.

Like our review, previous systematic reviews have found no evidence that financial incentives improve the quality of healthcare, with evidence of small benefits at best, and urged caution when policy makers are considering introducing new incentives [143, 144]. Although our study was not able to meta-analyse the effectiveness of quality improvement strategies, another review found the Plan-Do-Study-Act (PDSA) improved the quality of care in most included studies, despite many studies not adhering to the key method features [145, 146].

### Future implications

A limitation of this review was the quality of the included studies; many studies used a cluster design but did not account for clustering in their analysis. Studies should ensure that studies are powered to detect modest effects on behavioural outcomes, account for clustering and allow sufficient follow-up time to accrue sufficient events to detect changes in behavioural outcome as well as process outcomes.

No studies looked specifically at implementation strategies in remote consultations. As the delivery of primary care is changing, future research should consider whether these implementation strategies are effective across consultation modalities.

## Conclusions

These results show that a broad suite of intervention strategies, and in particular multicomponent interventions may improve processes of preventive care. However, there is no evidence that these strategies improve patient outcomes through behaviour change, such as smoking cessation, increased physical activity, or reduced bodyweight or alcohol consumption. There is evidence that introducing population health interventions affect individuals’ attempts to change their behaviour and the success of those attempts to change [147, 148]. Consequently, it may be helpful for policy makers to combine multicomponent implementation strategies to increase the delivery of preventive healthcare in primary care with population-level interventions to maximise health benefits.

## Supplementary Information

Additional file 1: Database search strategy.

SAdditional file 2: Imputed calculations.

Additional file 3: Figures S1-S7. FigS1 – Meta-analysis summary diamonds as random effects (main analysis), Hartung-Knapp-Sidik-Jonkman (HKSJ) and inverse variance heterogeneity (IVhet) models for process outcomes. FigS2 - Meta-analysis summary diamonds as random effects (main analysis), Hartung-Knapp-Sidik-Jonkman (HKSJ) and inverse variance heterogeneity model (IVhet) models for behavioural outcomes. FigS3 - Random effects meta-analysis summary diamonds excluding studies at high risk of bias and those that required data imputation for process outcomes. FigS4 - Random effects meta-analysis summary diamonds excluding studies at high risk of bias and those that required data imputation for behavioural outcomes. FigS5 - Random effects meta-analysis of behavioural outcomes, excluding Moore 2003, Welzel 2021 and Goodfellow 2016. FigS6 - Random effects meta-analysis summary diamonds of health behaviour subgroups for process outcomes. FigS7 - Random effects meta-analysis summary diamonds of health behaviour subgroups for behavioural outcomes.

Additional file 4: Stata code.

Additional file 5: Table S1 - Characteristics of included studies.

Additional file 6: PRISMA 2020 for Abstracts Checklist.

Additional file 7: PRISMA 2020 Checklist.

Additional file 8: Table S1 - Prediction interval calculation and interpretation.

## Data Availability

All data generated or analysed during this study are included in this published article and its additional files.

## Abbreviations

QI: Quality improvement
PROSPERO: International Prospective Register of Systematic Reviews
EPOC: Effective Practice and Organisation of Care
PRISMA: Preferred Reporting Items for Systematic reviews and Meta-Analysis
CINAHL: Cumulated Index in Nursing and Allied Health Literature
CENTRAL: The Cochrane Central Register of Controlled Trials
Embase: Excerpta Medica Database
PMC: PubMed Central
MEDLINE: Medical Literature Analysis and Retrieval System Online
NHS: National Health Service
cRT: Cluster randomised trial
cNRT: Cluster non-randomised trial
RT: Randomised trial
CBA: Controlled before-after
ITS: Interrupted time series
OR: Odds ratio
ICC: Intracluster correlation coefficient
HKSJ: Hartung-Knapp-Sidik-Jonkman
CI: Confidence interval
IVhet: Inverse variance heterogeneity
BMI: Body mass index
AUDIT: Alcohol Use Disorders Identification Test
PDSA: Plan-Do-Study-Act
