# Supplementary material for: Strategies to improve the implementation of preventive care in primary care: a systematic review and meta-analysis
Source: BMC Med. 2024 Sep 27;22:412. doi: 10.1186/s12916-024-03588-5 (PMC11437661; doi:10.1186/s12916-024-03588-5)
Supplement: Supplementary file 1 — Additional file 1: Database search strategy. [file 12916_2024_3588_MOESM1_ESM.docx]

*Supplementary Material – Search strategies*

Medline Search Strategy

| 1 | Primary Health Care/ |
| --- | --- |
| 2 | exp General Practice/ |
| 3 | general practitioners/ or physicians, family/ or physicians, primary care/ |
| 4 | exp Community Health Nursing/ |
| 5 | Nurses, Community Health/ |
| 6 | house calls/ or office visits/ |
| 7 | Community Health Services/ or Community Pharmacy Services/ or Community Health Centers/ |
| 8 | (primary adj2 (health* or care)).ti,ab,kf. |
| 9 | ((general or family) adj2 (pract* or doctor* or physician*)).ti,ab,kf. |
| 10 | family medicine.ti,ab,kf. |
| 11 | ((community adj2 nurs*) or health visitor? or district nurs*).ti,ab,kf. |
| 12 | (community adj2 (care or health* or service*)).ti,ab,kf. |
| 13 | (community adj2 pharmac*).ti,ab,kf. |
| 14 | ((home or house) adj (call? or visit?)).ti,ab,kf. |
| 15 | 1 or 2 or 3 or 4 or 5 or 6 or 7 or 8 or 9 or 10 or 11 or 12 or 13 or 14 |
| 16 | smoking cessation/ or smoking reduction/ or "tobacco use cessation"/ |
| 17 | healthy people programs/ or weight reduction programs/ |
| 18 | ((obes* or overweight or weight or diet* or nutrition*) adj3 (intervention? or program* or referral?)).ti,ab,kf. |
| 19 | (smoking adj3 (intervention? or program* or referral?)).ti,ab,kf. |
| 20 | ((physical activit* or exercis* or pa or walking or running or cycling or bicycl* or jog* or swim*) adj3 (intervention? or program* or referral? or prescri*)).ti,ab,kf. |
| 21 | ((alcohol* or drinking) adj3 (intervention? or program* or referral?)).ti,ab,kf. |
| 22 | Preventive Health Services/ |
| 23 | health education/ or health promotion/ |
| 24 | counseling/ or directive counseling/ or motivational interviewing/ |
| 25 | (intervention? or education or promotion or program*).ti. |
| 26 | ((education* or promot*) adj3 intervention?).ti,ab,kf. |
| 27 | (counsel* or advi?e or coach*).ti,ab,kf. |
| 28 | ((motivational or brief) adj2 (intervention? or advi?e or interview* or messag*)).ti,ab,kf. |
| 29 | 22 or 23 or 24 or 25 or 26 or 27 or 28 |
| 30 | smoking/ or exp "tobacco use"/ or Electronic Nicotine Delivery Systems/ |
| 31 | weight loss/ or exp overweight/ |
| 32 | exp Exercise/ or exp Sports/ or Motor Activity/ or Dancing/ |
| 33 | alcohol abstinence/ or exp alcohol drinking/ |
| 34 | exp Life Style/ |
| 35 | (smoker? or smoking or "tobacco use").ti,ab,kf. |
| 36 | (obes* or overweight).ti. or (weight adj2 (loss or lose or losing or control or manage* or reduc* or maintain or maintenance)).ti,ab,kf. |
| 37 | (physical activity or exercise?).ti,ab,kf. |
| 38 | alcohol*.ti. or (drinker? or (alcohol adj2 (risk* or "use" or consum* or drink* or abstain* or abstinence or reduc*))).ti,ab,kf. |
| 39 | ((healthy or healthful or healthier) adj2 (diet? or eating or lifestyle? or living or behavi*)).ti,ab,kf. |
| 40 | 30 or 31 or 32 or 33 or 34 or 35 or 36 or 37 or 38 or 39 |
| 41 | 29 and 40 |
| 42 | 16 or 17 or 18 or 19 or 20 or 21 or 41 |
| 43 | Reminder Systems/ |
| 44 | Physician Incentive Plans/ |
| 45 | reimbursement mechanisms/ or reimbursement, incentive/ |
| 46 | exp education, continuing/ or mentoring/ or exp inservice training/ |
| 47 | Quality Improvement/ |
| 48 | Total Quality Management/ |
| 49 | medical records systems, computerized/ or exp electronic health records/ |
| 50 | exp Health Personnel/ed |
| 51 | Decision Making, Computer-Assisted/ or Decision Support Techniques/ |
| 52 | exp Professional Role/ |
| 53 | (remider? or prompt? or alert?).ti,ab,kf. |
| 54 | (reimburse* or incentive? or reward?).ti. |
| 55 | ((financial or monetary) adj2 (reimburse* or incentive? or reward?)).ti,ab,kf. |
| 56 | ((quality or performance or benchmark*) adj2 (reimburse* or incentive? or reward?)).ti,ab,kf. |
| 57 | ((physician* or doctor? or general practi* or clinician? or nurse? or staff or personnel or professional? or provider?) adj3 (reimburse* or incentive? or reward?)).ti,ab,kf. |
| 58 | ("pay for quality" or "pay for performance" or p4q or p4p).ti,ab,kf. |
| 59 | ((quality based or performance based or result? based or output? based) adj2 (financ* or pay* or subsid*)).ti,ab,kf. |
| 60 | (quality improvement or total quality management).ti,ab,kf. |
| 61 | ((teach* or educat* or train* or mentor*) adj3 (physician* or doctor? or general practi* or clinician? or nurse? or staff or personnel or professional? or provider?)).ti,ab,kf. |
| 62 | (("in house" or "in service" or inservice or "in practice") adj2 training).ti,ab,kf. |
| 63 | (skill? adj2 (build* or development)).ti,ab,kf. |
| 64 | ((staff or professional) adj development).ti,ab,kf. |
| 65 | ((education* or academic*) adj2 (outreach or detailing)).ti,ab,kf. |
| 66 | ((audit or performance) adj2 feedback).ti,ab,kf. |
| 67 | ((electronic adj2 record?) or ehr or emr or epr).ti,ab,kf. |
| 68 | (mentor? or expert? or leader? or champion? or facilitator?).ti. |
| 69 | ((clinical or practice or health) adj2 (leader* or expert? or mentor* or champion* or facilitator?)).ti,ab,kf. |
| 70 | (role? adj2 (change? or changing or expand* or expansion)).ti,ab,kf. |
| 71 | 43 or 44 or 45 or 46 or 47 or 48 or 49 or 50 or 51 or 52 or 53 or 54 or 55 or 56 or 57 or 58 or 59 or 60 or 61 or 62 or 63 or 64 or 65 or 66 or 67 or 68 or 69 or 70 |
| 72 | randomized controlled trial.pt. |
| 73 | controlled clinical trial.pt. |
| 74 | multicenter study.pt. |
| 75 | pragmatic clinical trial.pt. |
| 76 | (randomis* or randomiz* or randomly).ti,ab. |
| 77 | groups.ab. |
| 78 | (trial or multicenter or multi center or multicentre or multi centre).ti. |
| 79 | (intervention? or effect? or impact? or controlled or control group? or (before adj5 after) or (pre adj5 post) or ((pretest or pre test) and (posttest or post test)) or quasiexperiment* or quasi experiment* or pseudo experiment* or pseudoexperiment* or evaluat* or time series or time point? or repeated measur*).ti,ab. |
| 80 | non-randomized controlled trials as topic/ |
| 81 | interrupted time series analysis/ |
| 82 | controlled before-after studies/ |
| 83 | or/72-82 |
| 84 | exp animals/ |
| 85 | humans/ |
| 86 | 84 not (84 and 85) |
| 87 | review.pt. |
| 88 | meta analysis.pt. |
| 89 | news.pt. |
| 90 | comment.pt. |
| 91 | editorial.pt. |
| 92 | cochrane database of systematic reviews.jn. |
| 93 | comment on.cm. |
| 94 | (systematic review or literature review).ti. |
| 95 | or/86-94 |
| 96 | 83 not 95 |
| 97 | 15 and 42 and 71 and 96 |

Embase Search Strategy

| 1 | primary medical care/ or primary health care/ | 192943 |
| --- | --- | --- |
| 2 | general practice/ | 81885 |
| 3 | general practitioner/ | 110449 |
| 4 | community health nursing/ | 23951 |
| 5 | health visitor/ | 1686 |
| 6 | home visit/ | 4243 |
| 7 | community care/ or health center/ | 95262 |
| 8 | community pharmacist/ | 1930 |
| 9 | (primary adj2 (health* or care)).ti,ab,kf. | 228793 |
| 10 | ((general or family) adj2 (pract* or doctor* or physician*)).ti,ab,kf. | 163110 |
| 11 | family medicine.ti,ab,kf. | 16222 |
| 12 | ((community adj2 nurs*) or health visitor? or district nurs*).ti,ab,kf. | 13137 |
| 13 | (community adj2 (care or health* or service*)).ti,ab,kf. | 89745 |
| 14 | (community adj2 pharmac*).ti,ab,kf. | 17228 |
| 15 | ((home or house) adj (call? or visit?)).ti,ab,kf. | 12648 |
| 16 | 1 or 2 or 3 or 4 or 5 or 6 or 7 or 8 or 9 or 10 or 11 or 12 or 13 or 14 or 15 | 646472 |
| 17 | smoking cessation program/ or smoking cessation/ or smoking reduction/ | 67251 |
| 18 | weight loss program/ | 2918 |
| 19 | ((obes* or overweight or weight or diet* or nutrition*) adj3 (intervention? or program* or referral?)).ti,ab,kf. | 73975 |
| 20 | (smoking adj3 (intervention? or program* or referral?)).ti,ab,kf. | 11966 |
| 21 | ((physical activit* or exercis* or pa or walking or running or cycling or bicycl* or jog* or swim*) adj3 (intervention? or program* or referral? or prescri*)).ti,ab,kf. | 68043 |
| 22 | ((alcohol* or drinking) adj3 (intervention? or program* or referral?)).ti,ab,kf. | 8709 |
| 23 | preventive health service/ | 29605 |
| 24 | health education/ or nutrition education/ or patient education/ or health promotion/ | 316983 |
| 25 | counseling/ or directive counseling/ or e-counseling/ or motivational interviewing/ or nutritional counseling/ or patient counseling/ or patient guidance/ | 135127 |
| 26 | (intervention? or education or promotion or program*).ti. | 626844 |
| 27 | ((education* or promot*) adj3 intervention?).ti,ab,kf. | 52628 |
| 28 | (counsel* or advi?e or coach*).ti,ab,kf. | 298043 |
| 29 | ((motivational or brief) adj2 (intervention? or advi?e or interview* or messag*)).ti,ab,kf. | 18734 |
| 30 | 23 or 24 or 25 or 26 or 27 or 28 or 29 | 1210107 |
| 31 | exp smoking/ or "tobacco use"/ or electronic cigarette/ | 449476 |
| 32 | body weight loss/ or exp *obesity/ | 329390 |
| 33 | exp exercise/ or exp physical activity/ | 803583 |
| 34 | alcohol consumption/ or alcohol abstinence/ or drinking behavior/ | 188979 |
| 35 | exp LifeStyle/ | 149943 |
| 36 | (smoker? or smoking or "tobacco use").ti,ab,kf. | 439313 |
| 37 | (obes* or overweight).ti. or (weight adj2 (loss or lose or losing or control or manage* or reduc* or maintain or maintenance)).ti,ab,kf. | 385696 |
| 38 | (physical activity or exercise?).ti,ab,kf. | 586127 |
| 39 | alcohol*.ti. or (drinker? or (alcohol adj2 (risk* or "use" or consum* or drink* or abstain* or abstinence or reduc*))).ti,ab,kf. | 277042 |
| 40 | ((healthy or healthful or healthier) adj2 (diet? or eating or lifestyle? or living or behavi*)).ti,ab,kf. | 48107 |
| 41 | 31 or 32 or 33 or 34 or 35 or 36 or 37 or 38 or 39 or 40 | 2241302 |
| 42 | 30 and 41 | 206283 |
| 43 | 17 or 18 or 19 or 20 or 21 or 22 or 42 | 350163 |
| 44 | Reminder System/ | 2936 |
| 45 | economic incentive/ or incentive/ or monetary reward/ | 2711 |
| 46 | reimbursement/ | 62403 |
| 47 | academic advisement/ or continuing education/ or education program/ or in service training/ or mentoring/ | 103437 |
| 48 | total quality management/ | 80169 |
| 49 | electronic health record/ | 29551 |
| 50 | decision support system/ or clinical decision support system/ | 29914 |
| 51 | (remider? or prompt? or alert?).ti,ab,kf. | 167802 |
| 52 | (reimburse* or incentive? or reward?).ti. | 29237 |
| 53 | ((financial or monetary) adj2 (reimburse* or incentive? or reward?)).ti,ab,kf. | 11262 |
| 54 | ((quality or performance or benchmark*) adj2 (reimburse* or incentive? or reward?)).ti,ab,kf. | 2083 |
| 55 | ((physician* or doctor? or general practi* or clinician? or nurse? or staff or personnel or professional? or provider?) adj3 (reimburse* or incentive? or reward?)).ti,ab,kf. | 5812 |
| 56 | ("pay for quality" or "pay for performance" or p4q or p4p).ti,ab,kf. | 3241 |
| 57 | ((quality based or performance based or result? based or output? based) adj2 (financ* or pay* or subsid*)).ti,ab,kf. | 609 |
| 58 | (quality improvement or total quality management).ti,ab,kf. | 76325 |
| 59 | ((teach* or educat* or train* or mentor*) adj3 (physician* or doctor? or general practi* or clinician? or nurse? or staff or personnel or professional? or provider?)).ti,ab,kf. | 177583 |
| 60 | (("in house" or "in service" or inservice or "in practice") adj2 training).ti,ab,kf. | 2963 |
| 61 | (skill? adj2 (build* or development)).ti,ab,kf. | 8660 |
| 62 | ((staff or professional) adj development).ti,ab,kf. | 17390 |
| 63 | ((education* or academic*) adj2 (outreach or detailing)).ti,ab,kf. | 3405 |
| 64 | ((audit or performance) adj2 feedback).ti,ab,kf. | 5417 |
| 65 | ((electronic adj2 record?) or ehr or emr or epr).ti,ab,kf. | 139776 |
| 66 | (mentor? or expert? or leader? or champion? or facilitator?).ti. | 53363 |
| 67 | ((clinical or practice or health) adj2 (leader* or expert? or mentor* or champion* or facilitator?)).ti,ab,kf. | 22198 |
| 68 | (role? adj2 (change? or changing or expand* or expansion)).ti,ab,kf. | 14154 |
| 69 | 44 or 45 or 46 or 47 or 48 or 49 or 50 or 51 or 52 or 53 or 54 or 55 or 56 or 57 or 58 or 59 or 60 or 61 or 62 or 63 or 64 or 65 or 66 or 67 or 68 | 881021 |
| 70 | randomized controlled trial/ | 715261 |
| 71 | controlled clinical trial/ | 465964 |
| 72 | quasi experimental study/ | 9658 |
| 73 | pretest posttest control group design/ | 600 |
| 74 | time series analysis/ | 32825 |
| 75 | experimental design/ | 22392 |
| 76 | multicenter study/ | 327367 |
| 77 | (randomis* or randomiz* or randomly).ti,ab. | 1472491 |
| 78 | groups.ab. | 3306085 |
| 79 | (trial or multicentre or multicenter or multi centre or multi center).ti. | 445838 |
| 80 | (intervention? or effect? or impact? or controlled or control group? or (before adj5 after) or (pre adj5 post) or ((pretest or pre test) and (posttest or post test)) or quasiexperiment* or quasi experiment* or pseudo experiment* or pseudoexperiment* or evaluat* or time series or time point? or repeated measur*).ti,ab. | 1.4E+07 |
| 81 | or/70-80 | 1.6E+07 |
| 82 | (systematic review or literature review).ti. | 275580 |
| 83 | cochrane database of systematic reviews.jn. | 16552 |
| 84 | exp animals/ or exp invertebrate/ or animal experiment/ or animal model/ or animal tissue/ or animal cell/ or nonhuman/ | 3.1E+07 |
| 85 | human/ or normal human/ or human cell/ | 2.4E+07 |
| 86 | 84 not (84 and 85) | 6939490 |
| 87 | 82 or 83 or 86 | 7228697 |
| 88 | 81 not 87 | 1.2E+07 |
| 89 | 16 and 43 and 69 and 88 | 4129 |

PsychINFO Search Strategy

| 1 | primary health care/ | 20175 |
| --- | --- | --- |
| 2 | family medicine/ | 1307 |
| 3 | family physicians/ or general practitioners/ | 7716 |
| 4 | Community Health/ or Community Services/ | 21046 |
| 5 | (primary adj2 (health* or care)).ti,ab,id. | 40927 |
| 6 | ((general or family) adj2 (pract* or doctor or physician*)).ti,ab,id. | 25497 |
| 7 | family medicine.ti,ab,id. | 2173 |
| 8 | ((community adj2 nurs*) or health visitor? or district nurs*).ti,ab,id. | 2795 |
| 9 | (community adj2 (care or health* or service*)).ti,ab,id. | 38497 |
| 10 | (community adj2 pharmac*).ti,ab,id. | 1163 |
| 11 | ((home or house) adj (call? or visit?)).ti,ab,id. | 3432 |
| 12 | 1 or 2 or 3 or 4 or 5 or 6 or 7 or 8 or 9 or 10 or 11 | 116074 |
| 13 | smoking cessation/ | 14388 |
| 14 | ((obes* or overweight or weight or diet* or nutrition*) adj3 (intervention? or program* or referral?)).ti,ab,id. | 11532 |
| 15 | (smoking adj3 (intervention? or program* or referral?)).ti,ab,id. | 5116 |
| 16 | ((physical activit* or exercis* or pa or walking or running or cycling or bicycl* or jog* or swim*) adj3 (intervention? or program* or referral? or prescri*)).ti,ab,id. | 13456 |
| 17 | ((alcohol* or drinking) adj3 (intervention? or program* or referral?)).ti,ab,id. | 7005 |
| 18 | preventive health services/ | 2568 |
| 19 | health education/ or health promotion/ | 39458 |
| 20 | counseling/ or motivational interviewing/ | 27629 |
| 21 | (intervention? or education or promotion or program*).ti. | 255528 |
| 22 | ((education* or promot*) adj3 intervention?).ti,ab,id. | 22487 |
| 23 | (counsel* or advi?e or coach*).ti,ab,id. | 161155 |
| 24 | ((motivational or brief) adj2 (intervention? or advi?e or interview* or messag*)).ti,ab,id. | 11759 |
| 25 | 18 or 19 or 20 or 21 or 22 or 23 or 24 | 440817 |
| 26 | tobacco smoking/ or electronic cigarettes/ | 35449 |
| 27 | weight loss/ or obesity/ or overweight/ | 30976 |
| 28 | exp exercise/ or physical activity/ | 48255 |
| 29 | drinking behavior/ or exp alcohol drinking patterns/ | 30222 |
| 30 | exp LifeStyle/ | 13213 |
| 31 | (smoker? or smoking or "tobacco use").ti,ab,id. | 60511 |
| 32 | (obes* or overweight).ti. or (weight adj2 (loss or lose or losing or control or manage* or reduc* or maintain or maintenance)).ti,ab,id. | 34665 |
| 33 | (physical activity or exercise?).ti,ab,id. | 103207 |
| 34 | alcohol*.ti. or (drinker? or (alcohol adj2 (risk* or "use" or consum* or drink* or abstain* or abstinence or reduc*))).ti,ab,id. | 95858 |
| 35 | ((healthy or healthful or healthier) adj2 (diet? or eating or lifestyle? or living or behavi*)).ti,ab,id. | 12822 |
| 36 | 26 or 27 or 28 or 29 or 30 or 31 or 32 or 33 or 34 or 35 | 298380 |
| 37 | 25 and 36 | 47063 |
| 38 | 13 or 14 or 15 or 16 or 17 or 37 | 75024 |
| 39 | exp incentives/ | 9909 |
| 40 | exp continuing education/ or mentor/ or educational programs/ | 40848 |
| 41 | electronic health records/ | 1087 |
| 42 | decision support systems/ | 3580 |
| 43 | (remider? or prompt? or alert?).ti,ab,id. | 21914 |
| 44 | (reimburse* or incentive? or reward?).ti. | 16839 |
| 45 | ((financial or monetary) adj2 (reimburse* or incentive? or reward?)).ti,ab,id. | 5652 |
| 46 | ((quality or performance or benchmark*) adj2 (reimburse* or incentive? or reward?)).ti,ab,id. | 1456 |
| 47 | ((physician* or doctor? or general practi* or clinician? or nurse? or staff or personnel or professional? or provider?) adj3 (reimburse* or incentive? or reward?)).ti,ab,id. | 1046 |
| 48 | ("pay for quality" or "pay for performance" or p4q or p4p).ti,ab,id. | 803 |
| 49 | ((quality based or performance based or result? based or output? based) adj2 (financ* or pay* or subsid*)).ti,ab,id. | 199 |
| 50 | (quality improvement or total quality management).ti,ab,id. | 6554 |
| 51 | ((teach* or educat* or train* or mentor*) adj3 (physician* or doctor? or general practi* or clinician? or nurse? or staff or personnel or professional? or provider?)).ti,ab,id. | 72908 |
| 52 | (("in house" or "in service" or inservice or "in practice") adj2 training).ti,ab,id. | 2712 |
| 53 | (skill? adj2 (build* or development)).ti,ab,id. | 9795 |
| 54 | ((staff or professional) adj development).ti,ab,id. | 26995 |
| 55 | ((education* or academic*) adj2 (outreach or detailing)).ti,ab,id. | 787 |
| 56 | ((audit or performance) adj2 feedback).ti,ab,id. | 3595 |
| 57 | ((electronic adj2 record?) or ehr or emr or epr).ti,ab,id. | 6144 |
| 58 | (mentor? or expert? or leader? or champion? or facilitator?).ti. | 24901 |
| 59 | ((clinical or practice or health) adj2 (leader* or expert? or mentor* or champion* or facilitator?)).ti,ab,id. | 6872 |
| 60 | (role? adj2 (change? or changing or expand* or expansion)).ti,ab,id. | 6310 |
| 61 | 39 or 40 or 41 or 42 or 43 or 44 or 45 or 46 or 47 or 48 or 49 or 50 or 51 or 52 or 53 or 54 or 55 or 56 or 57 or 58 or 59 or 60 | 233635 |
| 62 | (clinical trial or empirical study or experimental replication or followup study or longitudinal study or prospective study or quantitative study or treatment outcome).md. | 2774609 |
| 63 | experimental design/ | 12234 |
| 64 | between groups design/ | 562 |
| 65 | quantitative methods/ | 3788 |
| 66 | quasi experimental methods/ | 427 |
| 67 | (randomised or randomized or randomly or controlled or control group? or evaluat* or time series or time point or time points or quasi experiment* or quasiexperiment* or (before adj5 after) or (pre adj5 post) or ((pretest or pre test) and (posttest or post test)) or multicenter study or multicentre study or multi center study or multi centre study or repeated measur*).ti,ab. | 915321 |
| 68 | (trial or effect? or impact? or intervention?).ti. | 513967 |
| 69 | exp clinical trial/ | 13274 |
| 70 | ((clinical or control*) adj3 trial*).ti,ab. | 86854 |
| 71 | ((singl* or doubl* or trebl* or tripl*) adj5 (blind* or mask*)).ti,ab. | 28459 |
| 72 | (volunteer* or control group or controls).ti,ab. | 268969 |
| 73 | placebo/ or placebo*.ti,ab. | 43017 |
| 74 | pretesting/ | 410 |
| 75 | posttesting/ | 547 |
| 76 | repeated measures/ | 708 |
| 77 | time series/ | 2564 |
| 78 | or/62-77 | 3211295 |
| 79 | 12 and 38 and 61 and 78 | 775 |

CINAHL Search Strategy

| S13 | S4 OR S5 OR S12 |
| --- | --- |
| S14 | (MH "Reminder Systems") OR (MH "Physician Incentive Plans") OR (MH "Reimbursement Mechanisms") OR (MH "Reimbursement, Incentive") OR (MH "Employee Incentive Programs") OR (MM "Education, Continuing") OR (MH "Staff Development") OR (MH "Mentorship") OR (MH "Quality Improvement") OR (MH "Electronic Health Records") OR (MH "Decision Making, Computer Assisted") OR (MH "Decision Support Techniques") |
| S15 | ( ((TI remider# OR AB remider# OR SU remider#) OR (TI prompt# OR AB prompt# OR SU prompt#) OR (TI alert# OR AB alert# OR SU alert#)) ) OR ( (TI reimburse* OR TI incentive# OR TI reward#) ) OR ( (((TI financial OR AB financial OR SU financial) OR (TI monetary OR AB monetary OR SU monetary)) N2 ((TI reimburse* OR AB reimburse* OR SU reimburse*) OR (TI incentive# OR AB incentive# OR SU incentive#) OR (TI reward# OR AB reward# OR SU reward#))) ) OR ( (((TI quality OR AB quality OR SU quality) OR (TI performance OR AB performance OR SU performance) OR (TI benchmark* OR AB benchmark* OR SU benchmark*)) N2 ((TI reimburse* OR AB reimburse* OR SU reimburse*) OR (TI incentive# OR AB incentive# OR SU incentive#) OR (TI reward# OR AB reward# OR SU reward#))) ) OR ( (((TI physician* OR AB physician* OR SU physician*) OR (TI doctor# OR AB doctor# OR SU doctor#) OR (TI "general practi*" OR AB "general practi*" OR SU "general practi*") OR (TI clinician# OR AB clinician# OR SU clinician#) OR (TI nurse# OR AB nurse# OR SU nurse#) OR (TI staff OR AB staff OR SU staff) OR (TI personnel OR AB personnel OR SU personnel) OR (TI professional# OR AB professional# OR SU professional#) OR (TI provider# OR AB provider# OR SU provider#)) N3 ((TI reimburse* OR AB reimburse* OR SU reimburse*) OR (TI incentive# OR AB incentive# OR SU incentive#) OR (TI reward# OR AB reward# OR SU reward#))) ) OR ( ((TI "pay for quality" OR AB "pay for quality" OR SU "pay for quality") OR (TI "pay for performance" OR AB "pay for performance" OR SU "pay for performance") OR (TI p4q OR AB p4q OR SU p4q) OR (TI p4p OR AB p4p OR SU p4p)) ) OR ( (((TI "quality based" OR AB "quality based" OR SU "quality based") OR (TI "performance based" OR AB "performance based" OR SU "performance based") OR (TI "result# based" OR AB "result# based" OR SU "result# based") OR (TI "output# based" OR AB "output# based" OR SU "output# based")) N2 ((TI financ* OR AB financ* OR SU financ*) OR (TI pay* OR AB pay* OR SU pay*) OR (TI subsid* OR AB subsid* OR SU subsid*))) ) OR ( ((TI "quality improvement" OR AB "quality improvement" OR SU "quality improvement") OR (TI "total quality management" OR AB "total quality management" OR SU "total quality management")) ) OR ( (((TI teach* OR AB teach* OR SU teach*) OR (TI educat* OR AB educat* OR SU educat*) OR (TI train* OR AB train* OR SU train*) OR (TI mentor* OR AB mentor* OR SU mentor*)) N3 ((TI physician* OR AB physician* OR SU physician*) OR (TI doctor# OR AB doctor# OR SU doctor#) OR (TI "general practi*" OR AB "general practi*" OR SU "general practi*") OR (TI clinician# OR AB clinician# OR SU clinician#) OR (TI nurse# OR AB nurse# OR SU nurse#) OR (TI staff OR AB staff OR SU staff) OR (TI personnel OR AB personnel OR SU personnel) OR (TI professional# OR AB professional# OR SU professional#) OR (TI provider# OR AB provider# OR SU provider#))) ) OR ( (((TI "in house" OR AB "in house" OR SU "in house") OR (TI "in service" OR AB "in service" OR SU "in service") OR (TI inservice OR AB inservice OR SU inservice) OR (TI "in practice" OR AB "in practice" OR SU "in practice")) N2 (TI training OR AB training OR SU training)) "2356" ((TI skill# OR AB skill# OR SU skill#) N2 ((TI build* OR AB build* OR SU build*) OR (TI development OR AB development OR SU development))) ) OR ( (((TI staff OR AB staff OR SU staff) OR (TI professional OR AB professional OR SU professional)) W1 (TI development OR AB development OR SU development)) ) OR ( (((TI education* OR AB education* OR SU education*) OR (TI academic* OR AB academic* OR SU academic*)) N2 ((TI outreach OR AB outreach OR SU outreach) OR (TI detailing OR AB detailing OR SU detailing))) ) |
| S16 | ( (((TI audit OR AB audit OR SU audit) OR (TI performance OR AB performance OR SU performance)) N2 (TI feedback OR AB feedback OR SU feedback)) ) OR ( (((TI electronic OR AB electronic OR SU electronic) N2 (TI record# OR AB record# OR SU record#)) OR (TI ehr OR AB ehr OR SU ehr) OR (TI emr OR AB emr OR SU emr) OR (TI epr OR AB epr OR SU epr)) ) OR ( (TI mentor# OR TI expert# OR TI leader# OR TI champion# OR TI facilitator#) ) OR ( (((TI clinical OR AB clinical OR SU clinical) OR (TI practice OR AB practice OR SU practice) OR (TI health OR AB health OR SU health)) N2 ((TI leader* OR AB leader* OR SU leader*) OR (TI expert# OR AB expert# OR SU expert#) OR (TI mentor* OR AB mentor* OR SU mentor*) OR (TI champion* OR AB champion* OR SU champion*) OR (TI facilitator# OR AB facilitator# OR SU facilitator#))) ) OR ( ((TI role# OR AB role# OR SU role#) N2 ((TI change# OR AB change# OR SU change#) OR (TI changing OR AB changing OR SU changing) OR (TI expand* OR AB expand* OR SU expand*) OR (TI expansion OR AB expansion OR SU expansion))) ) |
| S17 | S14 OR S15 OR S16 |
| S18 | (MH "Clinical Trials") OR (MH "Randomized Controlled Trials") OR (MH "Controlled Before-After Studies") OR (MH "Interrupted Time Series Analysis") OR (MH "Nonrandomized Trials") OR (MH "Pretest-Posttest Design+") OR (MH "Intervention Trials") |
| S19 | ( ((TI randomis* OR AB randomis*) OR (TI randomiz* OR AB randomiz*) OR (TI randomly OR AB randomly)) ) OR AB groups OR ( (TI trial OR TI multicenter OR TI "multi center" OR TI multicentre OR TI "multi centre") ) OR ( ((TI intervention# OR AB intervention#) OR (TI effect# OR AB effect#) OR (TI impact# OR AB impact#) OR (TI controlled OR AB controlled) OR (TI "control group#" OR AB "control group#") OR ((TI before OR AB before) N5 (TI after OR AB after)) OR ((TI pre OR AB pre) N5 (TI post OR AB post)) OR (((TI pretest OR AB pretest) OR (TI "pre test" OR AB "pre test")) AND ((TI posttest OR AB posttest) OR (TI "post test" OR AB "post test"))) OR (TI quasiexperiment* OR AB quasiexperiment*) OR (TI "quasi experiment*" OR AB "quasi experiment*") OR (TI "pseudo experiment*" OR AB "pseudo experiment*") OR (TI pseudoexperiment* OR AB pseudoexperiment*) OR (TI evaluat* OR AB evaluat*) OR (TI "time series" OR AB "time series") OR (TI "time point#" OR AB "time point#") OR (TI "repeated measur*" OR AB "repeated measur*")) ) |
| S20 | S18 OR S19 |
| S21 | S3 AND S13 AND S17 AND S20 |

CENTRAL Search Strategy

| ID | Search |
| --- | --- |
| #1 | [mh ^"Primary Health Care"] OR [mh "General Practice"] OR [mh ^"physicians, family"] OR [mh ^"physicians, primary care"] OR [mh "Community Health Nursing"] OR [mh ^"office visits"] OR [mh ^"Community Pharmacy Services"] |
| #2 | (primary:ti,ab,kw NEAR/2 (health*:ti,ab,kw OR care:ti,ab,kw)) |
| #3 | ((general:ti,ab,kw OR family:ti,ab,kw) NEAR/2 (pract*:ti,ab,kw OR doctor*:ti,ab,kw OR physician*:ti,ab,kw)) |
| #4 | family medicine:ti,ab,kw |
| #5 | ((community:ti,ab,kw NEAR/2 nurs*:ti,ab,kw) OR ("health" NEXT visitor?):ti,ab,kw OR ("district" NEXT nurs*):ti,ab,kw) |
| #6 | (community:ti,ab,kw NEAR/2 (care:ti,ab,kw OR health*:ti,ab,kw OR service*:ti,ab,kw)) |
| #7 | (community:ti,ab,kw NEAR/2 pharmac*:ti,ab,kw) |
| #8 | ((home:ti,ab,kw OR house:ti,ab,kw) NEXT (call?:ti,ab,kw OR visit?:ti,ab,kw)) |
| #9 | #1 OR #2 OR #3 OR #4 OR #5 OR #6 OR #7 OR #8 |
| #10 | [mh ^"smoking cessation"] OR [mh ^"smoking reduction"] OR [mh ^"tobacco use cessation"] OR [mh ^"weight reduction programs"] |
| #11 | ((obes*:ti,ab,kw OR overweight:ti,ab,kw OR weight:ti,ab,kw OR diet*:ti,ab,kw OR nutrition*:ti,ab,kw) NEAR/3 (intervention?:ti,ab,kw OR program*:ti,ab,kw OR referral?:ti,ab,kw)) |
| #12 | (smoking:ti,ab,kw NEAR/3 (intervention?:ti,ab,kw OR program*:ti,ab,kw OR referral?:ti,ab,kw)) |
| #13 | ((("physical" NEXT activit*):ti,ab,kw OR exercis*:ti,ab,kw OR pa:ti,ab,kw OR walking:ti,ab,kw OR running:ti,ab,kw OR cycling:ti,ab,kw OR bicycl*:ti,ab,kw OR jog*:ti,ab,kw OR swim*:ti,ab,kw) NEAR/3 (intervention?:ti,ab,kw OR program*:ti,ab,kw OR referral?:ti,ab,kw OR prescri*:ti,ab,kw)) |
| #14 | ((alcohol*:ti,ab,kw OR drinking:ti,ab,kw) NEAR/3 (intervention?:ti,ab,kw OR program*:ti,ab,kw OR referral?:ti,ab,kw)) |
| #15 | [mh ^"Preventive Health Services"] OR [mh ^"health promotion"] OR [mh ^"directive counseling"] OR [mh ^"motivational interviewing"] |
| #16 | (intervention?:ti OR education:ti OR promotion:ti OR program*:ti) |
| #17 | ((education*:ti,ab,kw OR promot*:ti,ab,kw) NEAR/3 intervention?:ti,ab,kw) |
| #18 | (counsel*:ti,ab,kw OR advi?e:ti,ab,kw OR coach*:ti,ab,kw) |
| #19 | ((motivational:ti,ab,kw OR brief:ti,ab,kw) NEAR/2 (intervention?:ti,ab,kw OR advi?e:ti,ab,kw OR interview*:ti,ab,kw OR messag*:ti,ab,kw)) |
| #20 | #15 OR #16 OR #17 OR #18 OR #19 |
| #21 | [mh ^smoking] OR [mh "tobacco use"] OR [mh ^"Electronic Nicotine Delivery Systems"] OR [mh overweight] OR [mh Exercise] OR [mh Sports] OR [mh ^"Motor Activity"] OR [mh ^Dancing] OR [mh "alcohol drinking"] OR [mh "Life Style"] |
| #22 | (smoker?:ti,ab,kw OR smoking:ti,ab,kw OR "tobacco use":ti,ab,kw) |
| #23 | (obes*:ti OR overweight:ti) OR (weight:ti,ab,kw NEAR/2 (loss:ti,ab,kw OR lose:ti,ab,kw OR losing:ti,ab,kw OR control:ti,ab,kw OR manage*:ti,ab,kw OR reduc*:ti,ab,kw OR maintain:ti,ab,kw OR maintenance:ti,ab,kw)) |
| #24 | ("physical activity":ti,ab,kw OR exercise?:ti,ab,kw) |
| #25 | alcohol*:ti OR (drinker?:ti,ab,kw OR (alcohol:ti,ab,kw NEAR/2 (risk*:ti,ab,kw OR use:ti,ab,kw OR consum*:ti,ab,kw OR drink*:ti,ab,kw OR abstain*:ti,ab,kw OR abstinence:ti,ab,kw OR reduc*:ti,ab,kw))) |
| #26 | ((healthy:ti,ab,kw OR healthful:ti,ab,kw OR healthier:ti,ab,kw) NEAR/2 (diet?:ti,ab,kw OR eating:ti,ab,kw OR lifestyle?:ti,ab,kw OR living:ti,ab,kw OR behavi*:ti,ab,kw)) |
| #27 | #21 OR #22 OR #23 OR #24 OR #25 OR #26 |
| #28 | #20 AND #27 |
| #29 | #10 OR #11 OR #12 OR #13 OR #14 OR #28 |
| #30 | [mh ^"Reminder Systems"] OR [mh ^"reimbursement, incentive"] [mh "education, continuing"] OR [mh ^mentoring] OR [mh "inservice training"] OR [mh ^"Quality Improvement"] OR [mh "electronic health records"] OR [mh "Health Personnel"/ed] OR [mh ^"Decision Making, Computer-Assisted"] OR [mh ^"Decision Support Techniques"] [mh "Professional Role"] |
| #31 | (remider?:ti,ab,kw OR prompt?:ti,ab,kw OR alert?:ti,ab,kw) |
| #32 | (reimburse*:ti OR incentive?:ti OR reward?:ti) |
| #33 | ((financial:ti,ab,kw OR monetary:ti,ab,kw) NEAR/2 (reimburse*:ti,ab,kw OR incentive?:ti,ab,kw OR reward?:ti,ab,kw)) |
| #34 | ((quality:ti,ab,kw OR performance:ti,ab,kw OR benchmark*:ti,ab,kw) NEAR/2 (reimburse*:ti,ab,kw OR incentive?:ti,ab,kw OR reward?:ti,ab,kw)) |
| #35 | ((physician*:ti,ab,kw OR doctor?:ti,ab,kw OR ("general" NEXT practi*):ti,ab,kw OR clinician?:ti,ab,kw OR nurse?:ti,ab,kw OR staff:ti,ab,kw OR personnel:ti,ab,kw OR professional?:ti,ab,kw OR provider?:ti,ab,kw) NEAR/3 (reimburse*:ti,ab,kw OR incentive?:ti,ab,kw OR reward?:ti,ab,kw)) |
| #36 | ("pay for quality":ti,ab,kw OR "pay for performance":ti,ab,kw OR p4q:ti,ab,kw OR p4p:ti,ab,kw) |
| #37 | (("quality based":ti,ab,kw OR "performance based":ti,ab,kw OR (result? NEXT "based"):ti,ab,kw OR (output? NEXT "based"):ti,ab,kw) NEAR/2 (financ*:ti,ab,kw OR pay*:ti,ab,kw OR subsid*:ti,ab,kw)) |
| #38 | ("quality improvement":ti,ab,kw OR "total quality management":ti,ab,kw) |
| #39 | ((teach*:ti,ab,kw OR educat*:ti,ab,kw OR train*:ti,ab,kw OR mentor*:ti,ab,kw) NEAR/3 (physician*:ti,ab,kw OR doctor?:ti,ab,kw OR ("general" NEXT practi*):ti,ab,kw OR clinician?:ti,ab,kw OR nurse?:ti,ab,kw OR staff:ti,ab,kw OR personnel:ti,ab,kw OR professional?:ti,ab,kw OR provider?:ti,ab,kw)) |
| #40 | (("in house":ti,ab,kw OR "in service":ti,ab,kw OR inservice:ti,ab,kw OR "in practice":ti,ab,kw) NEAR/2 training:ti,ab,kw) |
| #41 | (skill?:ti,ab,kw NEAR/2 (build*:ti,ab,kw OR development:ti,ab,kw)) |
| #42 | ((staff:ti,ab,kw OR professional:ti,ab,kw) NEXT development:ti,ab,kw) |
| #43 | ((education*:ti,ab,kw OR academic*:ti,ab,kw) NEAR/2 (outreach:ti,ab,kw OR detailing:ti,ab,kw)) |
| #44 | ((audit:ti,ab,kw OR performance:ti,ab,kw) NEAR/2 feedback:ti,ab,kw) |
| #45 | ((electronic:ti,ab,kw NEAR/2 record?:ti,ab,kw) OR ehr:ti,ab,kw OR emr:ti,ab,kw OR epr:ti,ab,kw) or (mentor?:ti OR expert?:ti OR leader?:ti OR champion?:ti OR facilitator?:ti) |
| #46 | ((clinical:ti,ab,kw OR practice:ti,ab,kw OR health:ti,ab,kw) NEAR/2 (leader*:ti,ab,kw OR expert?:ti,ab,kw OR mentor*:ti,ab,kw OR champion*:ti,ab,kw OR facilitator?:ti,ab,kw)) |
| #47 | (role?:ti,ab,kw NEAR/2 (change?:ti,ab,kw OR changing:ti,ab,kw OR expand*:ti,ab,kw OR expansion:ti,ab,kw)) |
| #48 | #30 OR #31 OR #32 OR #33 OR #34 OR #35 OR #36 OR #37 OR #38 OR #39 OR #40 OR #41 OR #42 OR #43 OR #44 OR #45 OR #46 OR #47 |
| #49 | #9 AND #29 AND #48 |

Proquest Search Strategy

| S1 | [(TI,AB,IF(primary) NEAR/2 (TI,AB,IF(health*) OR TI,AB,IF(care))) OR ((TI,AB,IF(general) OR TI,AB,IF(family)) NEAR/2 (TI,AB,IF(pract*) OR TI,AB,IF(doctor*) OR TI,AB,IF(physician*))) OR TI,AB,IF("family medicine") OR ((TI,AB,IF(community) NEAR/2 TI,AB,IF(nurs*)) OR TI,AB,IF("health visitor?") OR TI,AB,IF("district nurs*")) OR (TI,AB,IF(community) NEAR/2 (TI,AB,IF(care) OR TI,AB,IF(health*) OR TI,AB,IF(service*))) OR (TI,AB,IF(community) NEAR/2 TI,AB,IF(pharmac*)) OR ((TI,AB,IF(home) OR TI,AB,IF(house)) PRE/0 (TI,AB,IF(call?) OR TI,AB,IF(visit?)))](https://www.proquest.com/recentsearches.recentsearchtabview.recentsearchesgridview.scrolledrecentsearchlist.checkdbssearchlink:rerunsearch/8781B74B37EF4AC5PQ/None?site=pqdtglobal&t:ac=RecentSearches) |
| --- | --- |
| S2 | [(obese:ti,ab,kw OR obesity:ti,ab,kw OR overweight:ti,ab,kw OR diet?:ti,ab,kw OR nutrition*.ti,ab,kw. OR smoking:ti,ab,kw OR ("physical" NEXT activit*):ti,ab,kw OR exercis*:ti,ab,kw OR pa:ti,ab,kw OR walking:ti,ab,kw OR running:ti,ab,kw OR cycling:ti,ab,kw OR bicycl*:ti,ab,kw OR jog*:ti,ab,kw OR swim*:ti,ab,kw OR alcohol*:ti,ab,kw OR drinking:ti,ab,kw OR "life style":ti,ab,kw) AND (intervention:ti,ab,kw OR interventions:ti,ab,kw OR program?:ti,ab,kw OR programme?:ti,ab,kw OR referral?:ti,ab,kw OR counseling:ti,ab,kw OR counselling:ti,ab,kw OR "motivational interviewing":ti,ab,kw OR advice:ti,ab,kw OR "health education":ti,ab,kw OR promotion:ti,ab,kw)](https://www.proquest.com/recentsearches.recentsearchtabview.recentsearchesgridview.scrolledrecentsearchlist.checkdbssearchlink:rerunsearch/96DC9BDC31874624PQ/None?site=pqdtglobal&t:ac=RecentSearches) |
| S3 | [reminder?:ti,ab,kw OR alert?:ti,ab,kw OR prompt?:ti,ab,kw OR reimbursement:ti,ab,kw OR incentive?:ti,ab,kw OR reward?:ti,ab,kw OR "educational outreach":ti,ab,kw OR "continuing education":ti,ab,kw OR training:ti,ab,kw OR "staff development":ti,ab,kw OR "professional development":ti,ab,kw OR "skills development":ti,ab,kw OR "academic detailing":ti,ab,kw OR "electronic health record?":ti,ab,kw OR "electronic medical record?":ti,ab,kw OR "electronic patient record?":ti,ab,kw OR emr:ti,ab,kw OR ehr:ti,ab,kw OR epr:ti,ab,kw OR "decision support":ti,ab,kw OR "quality improvement":ti,ab,kw OR "total quality management":ti,ab,kw OR leader?:ti,ab,kw OR mentor?:ti,ab,kw OR mentorship:ti,ab,kw OR champion?:ti,ab,kw OR facilitator?:ti,ab,kw](https://www.proquest.com/recentsearches.recentsearchtabview.recentsearchesgridview.scrolledrecentsearchlist.checkdbssearchlink:rerunsearch/2C339BCDE80249F7PQ/None?site=pqdtglobal&t:ac=RecentSearches) |
| S4 | S1 AND S2 AND S3 |

Europe PMC Search Strategy

| (ABSTRACT:"primary care" OR TITLE:"primary care" OR ABSTRACT:"general practi*" OR TITLE:"general practi*" OR ABSTRACT:"family medicine" OR TITLE:"family medicine" OR ABSTRACT:"physician*" OR TITLE:"physician*") AND (ABSTRACT:"lifestyle" OR TITLE:"lifestyle" OR ABSTRACT:"life style" OR TITLE:"life style") AND (ABSTRACT:"electronic record*" OR TITLE:"electronic record*"OR ABSTRACT:"ehr" OR TITLE:"ehr" OR ABSTRACT:"emr" OR TITLE:"emr" OR ABSTRACT:"epr" OR TITLE:"epr") AND (SRC:"PPR") |
| --- |
| (ABSTRACT:"primary care" OR TITLE:"primary care" OR ABSTRACT:"general practi*" OR TITLE:"general practi*" OR ABSTRACT:"family medicine" OR TITLE:"family medicine" OR ABSTRACT:"physician*" OR TITLE:"physician*") AND (ABSTRACT:"preventive health" OR TITLE:"preventive health") AND (ABSTRACT:"mentor*" OR TITLE:"mentor*"OR ABSTRACT:"leader*" OR TITLE:"leader*" OR ABSTRACT:"champion*" OR TITLE:"champion*" OR ABSTRACT:"facilitator*" OR TITLE:"facilitator*") AND (SRC:"PPR") |
| (ABSTRACT:"primary care" OR TITLE:"primary care" OR ABSTRACT:"general practi*" OR TITLE:"general practi*" OR ABSTRACT:"family medicine" OR TITLE:"family medicine" OR ABSTRACT:"physician*" OR TITLE:"physician*") AND (ABSTRACT:"smoking" OR TITLE:"smoking" OR ABSTRACT:"tobacco" OR TITLE:"tobacco") AND (ABSTRACT:"mentor*" OR TITLE:"mentor*"OR ABSTRACT:"leader*" OR TITLE:"leader*" OR ABSTRACT:"champion*" OR TITLE:"champion*" OR ABSTRACT:"facilitator*" OR TITLE:"facilitator*") AND (SRC:"PPR") |
| (ABSTRACT:"primary care" OR TITLE:"primary care" OR ABSTRACT:"general practi*" OR TITLE:"general practi*" OR ABSTRACT:"family medicine" OR TITLE:"family medicine" OR ABSTRACT:"physician*" OR TITLE:"physician*") AND (ABSTRACT:"alcohol" OR TITLE:"alcohol" OR ABSTRACT:"drinking" OR TITLE:"drinking") AND (ABSTRACT:"mentor*" OR TITLE:"mentor*"OR ABSTRACT:"leader*" OR TITLE:"leader*" OR ABSTRACT:"champion*" OR TITLE:"champion*" OR ABSTRACT:"facilitator*" OR TITLE:"facilitator*") AND (SRC:"PPR") |
| (ABSTRACT:"primary care" OR TITLE:"primary care" OR ABSTRACT:"general practi*" OR TITLE:"general practi*" OR ABSTRACT:"family medicine" OR TITLE:"family medicine" OR ABSTRACT:"physician*" OR TITLE:"physician*") AND (ABSTRACT:"obes*" OR TITLE:"obes*" OR ABSTRACT:"overweight" OR TITLE:"overweight") AND (ABSTRACT:"mentor*" OR TITLE:"mentor*"OR ABSTRACT:"leader*" OR TITLE:"leader*" OR ABSTRACT:"champion*" OR TITLE:"champion*" OR ABSTRACT:"facilitator*" OR TITLE:"facilitator*") AND (SRC:"PPR") |
| (ABSTRACT:"primary care" OR TITLE:"primary care" OR ABSTRACT:"general practi*" OR TITLE:"general practi*" OR ABSTRACT:"family medicine" OR TITLE:"family medicine" OR ABSTRACT:"physician*" OR TITLE:"physician*") AND (ABSTRACT:"weight" OR TITLE:"weight" OR ABSTRACT:"diet*" OR TITLE:"diet*") AND (ABSTRACT:"mentor*" OR TITLE:"mentor*"OR ABSTRACT:"leader*" OR TITLE:"leader*" OR ABSTRACT:"champion*" OR TITLE:"champion*" OR ABSTRACT:"facilitator*" OR TITLE:"facilitator*") AND (SRC:"PPR") |
| (ABSTRACT:"primary care" OR TITLE:"primary care" OR ABSTRACT:"general practi*" OR TITLE:"general practi*" OR ABSTRACT:"family medicine" OR TITLE:"family medicine" OR ABSTRACT:"physician*" OR TITLE:"physician*") AND (ABSTRACT:"physical activity" OR TITLE:"physical activity" OR ABSTRACT:"exercise" OR TITLE:"exercise") AND (ABSTRACT:"mentor*" OR TITLE:"mentor*"OR ABSTRACT:"leader*" OR TITLE:"leader*" OR ABSTRACT:"champion*" OR TITLE:"champion*" OR ABSTRACT:"facilitator*" OR TITLE:"facilitator*") AND (SRC:"PPR") |
| (ABSTRACT:"primary care" OR TITLE:"primary care" OR ABSTRACT:"general practi*" OR TITLE:"general practi*" OR ABSTRACT:"family medicine" OR TITLE:"family medicine" OR ABSTRACT:"physician*" OR TITLE:"physician*") AND (ABSTRACT:"lifestyle" OR TITLE:"lifestyle" OR ABSTRACT:"life style" OR TITLE:"life style") AND (ABSTRACT:"mentor*" OR TITLE:"mentor*"OR ABSTRACT:"leader*" OR TITLE:"leader*" OR ABSTRACT:"champion*" OR TITLE:"champion*" OR ABSTRACT:"facilitator*" OR TITLE:"facilitator*") AND (SRC:"PPR") |
| (ABSTRACT:"primary care" OR TITLE:"primary care" OR ABSTRACT:"general practi*" OR TITLE:"general practi*" OR ABSTRACT:"family medicine" OR TITLE:"family medicine" OR ABSTRACT:"physician*" OR TITLE:"physician*") AND (ABSTRACT:"preventive health" OR TITLE:"preventive health") AND (ABSTRACT:"quality improvement" OR TITLE:"quality improvement" OR ABSTRACT:"QI" OR TITLE:"QI" OR ABSTRACT:"total quality management" OR TITLE:"total quality management") AND (SRC:"PPR") |
| (ABSTRACT:"primary care" OR TITLE:"primary care" OR ABSTRACT:"general practi*" OR TITLE:"general practi*" OR ABSTRACT:"family medicine" OR TITLE:"family medicine" OR ABSTRACT:"physician*" OR TITLE:"physician*") AND (ABSTRACT:"smoking" OR TITLE:"smoking" OR ABSTRACT:"tobacco" OR "TITLE:"tobacco") AND (ABSTRACT:"quality improvement" OR TITLE:"quality improvement" OR ABSTRACT:"QI" OR TITLE:"QI" OR ABSTRACT:"total quality management" OR TITLE:"total quality management") AND (SRC:"PPR") |
| (ABSTRACT:"primary care" OR TITLE:"primary care" OR ABSTRACT:"general practi*" OR TITLE:"general practi*" OR ABSTRACT:"family medicine" OR TITLE:"family medicine" OR ABSTRACT:"physician*" OR TITLE:"physician*") AND (ABSTRACT:"alcohol" OR TITLE:"alcohol" OR ABSTRACT:"drinking" OR "TITLE:"drinking") AND (ABSTRACT:"quality improvement" OR TITLE:"quality improvement" OR ABSTRACT:"QI" OR TITLE:"QI" OR ABSTRACT:"total quality management" OR TITLE:"total quality management") AND (SRC:"PPR") |
| (ABSTRACT:"primary care" OR TITLE:"primary care" OR ABSTRACT:"general practi*" OR TITLE:"general practi*" OR ABSTRACT:"family medicine" OR TITLE:"family medicine" OR ABSTRACT:"physician*" OR TITLE:"physician*") AND (ABSTRACT:"obes*" OR TITLE:"obes*" OR ABSTRACT:"overweight" OR "TITLE:"overweight") AND (ABSTRACT:"quality improvement" OR TITLE:"quality improvement" OR ABSTRACT:"QI" OR TITLE:"QI" OR ABSTRACT:"total quality management" OR TITLE:"total quality management") AND (SRC:"PPR") |
| (ABSTRACT:"primary care" OR TITLE:"primary care" OR ABSTRACT:"general practi*" OR TITLE:"general practi*" OR ABSTRACT:"family medicine" OR TITLE:"family medicine" OR ABSTRACT:"physician*" OR TITLE:"physician*") AND (ABSTRACT:"weight" OR TITLE:"weight" OR ABSTRACT:"diet*" OR "TITLE:"diet*") AND (ABSTRACT:"quality improvement" OR TITLE:"quality improvement" OR ABSTRACT:"QI" OR TITLE:"QI" OR ABSTRACT:"total quality management" OR TITLE:"total quality management") AND (SRC:"PPR") |
| (ABSTRACT:"primary care" OR TITLE:"primary care" OR ABSTRACT:"general practi*" OR TITLE:"general practi*" OR ABSTRACT:"family medicine" OR TITLE:"family medicine" OR ABSTRACT:"physician*" OR TITLE:"physician*") AND (ABSTRACT:"physical activity" OR TITLE:"physical activity" OR ABSTRACT:"exercise" OR "TITLE:"exercise") AND (ABSTRACT:"quality improvement" OR TITLE:"quality improvement" OR ABSTRACT:"QI" OR TITLE:"QI" OR ABSTRACT:"total quality management" OR TITLE:"total quality management") AND (SRC:"PPR") |
| (ABSTRACT:"primary care" OR TITLE:"primary care" OR ABSTRACT:"general practi*" OR TITLE:"general practi*" OR ABSTRACT:"family medicine" OR TITLE:"family medicine" OR ABSTRACT:"physician*" OR TITLE:"physician*") AND (ABSTRACT:"lifestyle" OR TITLE:"lifestyle" OR ABSTRACT:"life style" OR "TITLE:"life style") AND (ABSTRACT:"quality improvement" OR TITLE:"quality improvement" OR ABSTRACT:"QI" OR TITLE:"QI" OR ABSTRACT:"total quality management" OR TITLE:"total quality management") AND (SRC:"PPR") |
| (ABSTRACT:"primary care" OR TITLE:"primary care" OR ABSTRACT:"general practi*" OR TITLE:"general practi*" OR ABSTRACT:"family medicine" OR TITLE:"family medicine" OR ABSTRACT:"physician*" OR TITLE:"physician*") AND (ABSTRACT:"brief intervention*" OR TITLE:"brief intervention*") AND (SRC:"PPR") |
| (ABSTRACT:"primary care" OR TITLE:"primary care" OR ABSTRACT:"general practi*" OR TITLE:"general practi*" OR ABSTRACT:"family medicine" OR TITLE:"family medicine" OR ABSTRACT:"physician*" OR TITLE:"physician*") AND (TITLE:"brief") AND (SRC:"PPR") |
