# Supplementary material for: Strategies to improve the implementation of preventive care in primary care: a systematic review and meta-analysis
Source: BMC Med. 2024 Sep 27;22:412. doi: 10.1186/s12916-024-03588-5 (PMC11437661; doi:10.1186/s12916-024-03588-5)
Supplement: Supplementary file 2 — SAdditional file 2: Imputed calculations. [file 12916_2024_3588_MOESM2_ESM.docx]

***Imputed calculations***

Where an intra-cluster coefficient (ICC) was not provided, the upper quartile of ICCs provided from the included studies was used:

| **Study** | **ICC** |
| --- | --- |
| Minian 2019 | 0.134 |
| Goodfellow 2016 | 0.094 |
| Bardach 2013 | 0.1 |
| Linder 2009 | 0.007 |
| Wadland 2007 | 0.902 |
| Kaner 2003 | 0.064 |
| Rothemich 2008 | 0.061 |
| Campbell-Scherer 2019 | 0.4 |
| Rosario 2022 | 0.81 |
| VanLieshout 2016 | 0.05 |
| Jumbe 2022 | 0.059 |
| Goodfellow 2016 | 0.003 |
| Haller 2016 | 0.026 |
| Lennox 1998 | 0.13 |
|  |  |
| Upper quartile | **0.133** |

| Study | Rationale | Calculation |
| --- | --- | --- |
| **Clinical Reminders** | | |
| 1. Banerjee 2019 | Randomised trial, gave 2x2 table, need OR and 95% CI | Stata code:  cci 38 220 11 228  **OR = 3.58 (95% CI 1.74 – 7.95)** |
| 1. Rothemich 2008 | ICC = 0.061 given  Adjusted p value given  No adjusted effect size  Methods taken from Cochrane Handbook 23.1.4.1 | Intervention: 181/561; 9 practices  Control: 172/588; 9 practices  ICC =0.061  Average cluster size = (561+588)/ (18) = 63.8  Design effect for the trial is:  1 +(M-1)ICC = 1 +(63.8-1) x 0.061 = 4.83  Intervention sample size  561/4.83 = **116**  Intervention cases  181/4.83= **37**  Control sample size  588/4.83 = **122**  Control cases  172/4.83= **36**  Stata code  cci 37 79 36 86  **OR = 1.12 (95% CI 0.62 – 2.02)**  **Without rounding: OR 1.15 (95% CI 0.66-2.00)** |
| 1. Milch 2004 | No adjustment for clustering | Combining both stamp and SAQ arms as both clinical reminders of differing intensity.  Data from table 2 and figure 3:  Clinician advice: 38% of 77 patients (stamp) 47% of 92 patients (SAQ) 20% of 238 patients (control)  = 29/77 + 43/92 = 72/169 intervention received clinical advice  48/238 control received clinical advice  Average cluster size  Intervention 7+7 =14 physicians  Control = 15 physicians  (169+238)/29 = 14.034  Design effect for the trial is:  1 +(M-1)ICC = 1 +(14.034-1) x 0.133 = 2.734  Intervention sample size  169/2.734= 62  Intervention cases  72/2.734= **26**  Control sample size  238/2.734= 87  Control cases  48/2.734= 18  **Stata code: cci 26 36 18 69**  **OR = 2.77 (95% CI 1.26 – 6.10)**  **Without rounding: OR = 2.94 (95% CI = 1.42 – 6.08)**  Smoking cessation outcomes:  SAQ: 14/46  Stamp: 2/45  Control 17/154  Intervention combined = 16/91  Average cluster size = (154+91)/29 = 8.448  Design effect for the trial is:  1 +(M-1)ICC = 1 +(8.448-1) x 0.133 = 1.991  Intervention sample size  91/1.991= **46**  Intervention cases  16/1.991= **8**  Control sample size  154/1.991= **77**  Control cases  17/1.991= **9**  **Stata code: cci 8 38 9 68**  **OR = 1.59 (95% CI 0.49 – 5.06)**  **Without rounding: OR = 1.72 (95% CI = 0.61 – 4.87)** |
| **Facilitated relay of information** | | |
| 1. Grant 2014 | Difference in difference, need OR  Cochrane Handbook section 10.6 | Need to find standard deviation (SD) to use Chinn formula. As this is not in Grant 2014 paper, use a value from similar paper. In this review, Goodfellow 2016 has a similar BMI (SD) of 30.2 (5.4). The SD for weight = 17.9kg, in lbs = 39lbs  Chinn 2000 formula:  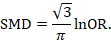  Effect size = EXP((3.1415*(**0.16/39**)/(SQRT(3))) = 1.34 = 1.0075  Lower CI = EXP((3.1415***0.10/39**)/(SQRT(3))) = 1.20 = 1.0047  Upper CI = EXP((3.1415***0.21/39**)/(SQRT(3))) = 1.46 = 1.0098 |
| 1. Saitz 2003 | Reasonable first order approximation to OR and CI | Using formula: p= k/n  SE(p) = SQRT((p(1-p))/ n)  P = 0.56  CI = 0.41-0.70  SE = (0.7-0.41)/ 3.92 = 0.07398  n = (0.56x 0.44)/ (0.07398)^2 = 45 therefore **25 events in sample of 45**  Similarly for other values :  P = 0.41; CI = 0.3-0.52  **31/ 77**  P = 0.22; CI = 0.13-0.35  **12/54**  P = 0.26; CI = 0.15-0.42  **11/41**  Calculating OR and CI for these values:  Process outcome OR = **1.85 (95% CI 0.98 – 3.90)**  **Without rounding: OR = 1.83 (95% CI = 0.87 – 3.85)**  Outcome outcome OR = **0.78 (95% CI 0.30 – 2.00)**  **Without rounding: OR = 0.80 (95% CI = 0.31 – 2.08)** |
| **Multicomponent Interventions** | | |
| 1. Ackerman 2005 | Give 2x2 table and adjusted p value for both process and clinical outcomes | Given exercise advice at provider visit, for all enrolled patients  p= 0.07 and therefore z = 1.8119 = logOR/ SE  OR = (45/127)/ (35/129) = 1.3060  logOR = 0.2669  SE = 0.2669/1.8119 = 0.1473  Log(95% CI) = logOR +/- 1.96* SE =0.2669 +/- (1.96*0.1473) = **0.5556, -0.0218**  e^0.5556= **1.74**  e^-0.0218= **0.98**  **aOR = 1.31 (95% CI 0.98-1.74)**  After 4 months regular exercise, for all enrolled patients  p= 0.06 and therefore z = 1.8808 = logOR/ SE  OR = (35/137)/ (28/136) = **1.2409**  logOR = 0.2158  SE = 0.2158/1.8808 = 0.1147  Log(95% CI) = logOR +/- 1.96* SE = 0.2158+/- (1.96 * 0.1147) = 0.4406, -0.00901  e^= **0.99**  e^= **1.55**  **aOR = 1.24 (95% CI 0.99 – 1.55)** |
| 1. Young 2002 | Give OR for posttest versus baseline for intervention and control groups. Need to have a between group comparison. | R code:  betaI<-log(1.68)  betaC<-log(1.23)  beta<-betaI-betaC  seI<-log(8.48/0.33)/3.92  seC<-log(7.48/0.20)/3.92  se<-sqrt(seI^2+seC^2)  beta  0.3117796  se  1.240736  exp(beta+1.96*se*c(0,-1,1))  1.3658537  0.1200241 15.5431866  Therefore **OR 1.37 (95% CI 0.12 – 15.54)** |
| 1. Twardella 2007 | Have 2x2 table (UC vs TI)for process outcome, need to adjust for clustering | The study gives 2x ICC in table 4. Use the largest of these to be conservative (0.042).  Intervention: 83/114; 21 medical practices  Control: 32/54; 20 medical practices  Average cluster size = 114+54/ 41 = 4.0976  Design effect for the trial is:  1 +(M-1)ICC = 1 +(4.0976-1) x 0.042 = 1.130  Intervention sample size  114/1.13= **101**  Intervention cases  83/1.13= **73**  Control sample size  54/1.13= **48**  Control cases  32/1.13= **28**  **Stata code: cci 73 28 28 20**  **OR = 1.86 (95% CI 0.85 –4.06)**  **Without rounding: OR = 1.84 (95% CI = 0.89 – 3.80)** |
| 1. Lee 2023 | Give log OR, SE, z, p value and OR in supplementary material | Need to calculate a CI for the OR  Log(95% CI) = logOR +/- 1.96* SE = 2.83+/- (1.96*0.11) = 3.05 and 2.61  e^3.05 = 21.02  e^2.61 = 13.66 |
| **Team Changes** | | |
| 1. Alageel 2019 | Have standardised mean difference, need effect measure | Mean difference (BMI) = -0.29  Standard deviation of control group BMI = 5.6  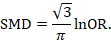  SMD = -0.29/5.6 = -0.0518  Upper CI = -0.31/5.6 = -0.0554  Lower CI = -0.27/5.6 = -0.0482  Effect size = EXP((3.1415*-**0.0518**)/(SQRT(3))) = 0.9103  Lower CI = EXP((3.1415*-**0.0482**)/(SQRT(3))) = 0.9044  Upper CI = EXP((3.1415*-**0.0554**)/(SQRT(3))) = 0.9163  Need to invert OR and CI  **OR = 1.10 (95% CI 1.09 – 1.11)** |
| 1. Schwartz 2014 | Have 2x2 table for smoking cessation outcomes, not adjusted for clustering | Use Control and Intervention (PMA support) arm.  Use table 3, self-reported quit for at least 1 week  Control: 63.8% of 141 = 90/141  PMA support: 64.1% of 92 =59/92  Average cluster size: (141+92)/14(teams in PMA + control) = 16.643  Design effect for the trial is:  1 +(M-1)ICC = 1 +(16.643 -1) x 0.133 = 3.081  Intervention sample size  92/3.081= **30**  Intervention cases  59/3.081= **19**  Control sample size  141/3.081= **46**  Control cases  90/3.081= **29**  **Stata code: cci 19 11 29 17**  **OR = 1.01 (95% CI 0.35 – 2.96)**  **Without rounding: OR = 1.01 (95% CI = 0.39 – 2.65)**  Prescribed Nicotine Replacement Therapy (from the VA’s EMR)  Calculate lost to follow up factor  83% followed up in control = 731 people  66% followed up in intervention = 278 people  6.2% control of 731 smokers  7.5% PMA intervention of 278 smokers  0.062 x 731 = 45/731  0.075 X 278 = 21/278  Average cluster size: (731+278)/14 = 72.071  Design effect for the trial is:  1 +(M-1)ICC = 1 +(72.071-1) x 0.133 = 10.452  Intervention sample size  278/10.452= **27**  Intervention cases  21/10.452= **2**  Control sample size  731/10.452= **70**  Control cases  45/10.452= **4**  **Stata code: cci 2 25 4 66**  **OR = 1.32 (95% CI 0.11 – 9.85)**  **Without rounding: OR = 1.25 (95% CI = 0.22 – 7.08)** |
| 1. Little 2009 | Have an adjusted p value, need to find a CI | Find an CI that is consistent with a p value of 0.01  p= 0.01 and therefore z = 2.5758 = logOR/ SE  OR = (2779/1249)/ (318/10282) = 71.94  logOR = 4.2758  SE =4.2758 /2.5758 = 1.660  Log(95% CI) = logOR +/- 1.96* SE = 4.2758+/- (1.96*1.660) = 7.5294 and 1.022  e^7.5294 = 1861.99  e^1.022= 2.779  **aOR = 71.94 (95% CI 2.78- 1862.00)** |
| **Clinician Education** | | |
| 1. Corelli 2022 |  | Peto method used to calculate OR  Peto method had to be used to manage the 0 non-events in the intervention arm. Using this method, p=0.01 which was a similar result to that reported in the paper (p = 0.001) with  **OR** **10.5 (95% CI 2.67-41.63)** |
| 1. Welzel 2021 | Have cohens d standardised effect sizes, can use Chinn 2000 formula to calculate OR from difference in difference | 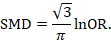  Process outcome  Effect size = EXP((3.1415***0.20**)/(SQRT(3))) = **1.44**  Lower CI = EXP((3.1415*-**0.18**)/(SQRT(3))) = **0.71**  Upper CI = EXP((3.1415***0.60**)/(SQRT(3))) = **2.92**  Clinical outcome NB the 6 month outcome was used here as the 12 month outcomes were incorrectly reported.  Effect size = EXP((3.1415*-**0.29**)/(SQRT(3))) = **0.59**  Lower CI = EXP((3.1415***-0.65**)/(SQRT(3))) = **0.31**  Upper CI = EXP((3.1415***0.77**)/(SQRT(3))) = **4.04** |
| 1. Malta 2016 | Unadjusted 2 x 2 table | Stata code:  Cci 82 58 47 94  OR = 2.83 (95% CI 1.69-7.73)  Adjust for clustering using the highest ICC in the included studies (to be conservative)  Imputed ICC (upper quartile from other studies) = 0.133  Intervention: 82/140; 9 practices  Control: 47/141; 8 practices  ICC =0.133  Average cluster size = (140+141)/ (17) = 16.5294  Design effect for the trial is:  1 +(M-1)ICC = 1 +(16.5294-1) x 0.133 = 3.065  Intervention sample size  140/3.065= **46**  Intervention cases  82/3.065= **27**  Control sample size  141/3.065= **46**  Control cases  47/3.065= **15**  **Stata code: cci 27 19 15 31**  **OR = 2.94 (95% CI 1.16 – 7.53)**  **Without rounding: OR = 2.82 (95% CI = 1.21 – 6.61)** |
| 1. Rosario 2022 | Have cohens d standardised effect size, can use Chinn’s formula to calculate OR | Brief intervention rate at 12 month follow up  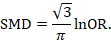  Process outcome  Effect size = EXP((3.1415***0.27**)/(SQRT(3))) = **1.63**  Lower CI = EXP((3.1415*-**0.52**)/(SQRT(3))) = **0.39**  Upper CI = EXP((3.1415***1.05**)/(SQRT(3))) = **6.77** |
| 1. Ruf 2010 | Gave an adjusted p value but no effect size | p= 0.781 and therefore z = 0.278 = logOR/ SE  OR = (27*5)/ (5*14) = **1.9286**  logOR = 0.6568  SE = 0.6568/ 0.278 = 2.3626  95% CI = logOR +/- 1.96* SE = 0.6568 +/- (1.96*2.3626) = **-3.97, 5.29**  e^-3.97 = **0.02**  e^5.29 = **197.80**  **aOR = 1.93 (95% CI 0.02-197.80)** |
| 1. Kaner 2003 | Table of results unadjusted for clustering, give ICC of 0.064 for similar studies. | We used the estimate given in the paper in the absence of an actual ICC for the study.  Intervention: 704/1130; 134 practices  Control: 222/370; 76 practices  ICC =0.064  Average cluster size = (1130+370)/ (134+76) = 7.1429  Design effect for the trial is:  1 +(M-1)ICC = 1 +(7.1429-1) x 0.064 = 1.3931  Intervention sample size  1130/1.3931= **811**  Intervention cases  704/1.3931= **505**  Control sample size  370/1.3931= **275**  Control cases  222/1.3931= **165**  **Stata code: cci 505 306 165 110**  **OR = 1.10 (95% CI 0.82– 1.47)**  **Without rounding: OR = 1.10 (95% CI = 0.83 – 1.46 )** |
| 1. Prokhorov 2010 | Give and adjusted p value and a 2x2 table | p= .04 and therefore z = 2.0537 = logOR/ SE  OR = (10/52)/ (6/51) = **1.6346**  logOR = 0.4914  SE = 0.4914/ 2.0537 = 0.2393  95% CI = logOR +/- 1.96* SE = 0.4914 +/- (1.96*0.2393) = **0.96, 0.0223**  e^0.96 = **2.61**  e^0.0223 = **1.02**  **aOR = 1.63 (95% CI 1.02-2.61)** |
| 1. Keller 2000 | Give stages of change table, need to calculate an effect measure | Create 2x2 table using those people who were not at motivation at baseline and then moved to motivation post intervention/ control  At baseline, not at motivation:  IG = 21+15+2+2 = 40  CG = 30 +17+6+3 = 56  Of these 5/40 moved to motivation in IG and 8/56 moved to motivation in C  Average cluster size = (40+56)/74 = 1.297  Use upper quartile ICC of 0.133 from other studies:  Design effect for the trial is:  1 +(M-1)ICC = 1 +(1.297-1) x 0.133 = **1.0395**  Intervention sample size  40/1.0395= **38**  Intervention cases  5/1.0395= **5**  Control sample size  56/1.0395= **54**  Control cases  8/1.0395= **8**  **Stata code: cci 5 33 8 46**  **OR = 0.87 (95% CI 0.21 – 3.35)**  **Without rounding: OR = 0.86 (95% CI = 0.25 – 2.91 )** |
| 1. Adams 1998 | Have % patients where provider tried to set up a follow up appointment, need an effect measure | Intervention = 67/100  Control = 6/100  p = 0.001 and therefore z = 3.2905= logOR/ SE  OR = (67/33)/ (6/94) = 31.81  logOR = 3.4597  SE = 3.4597/3.2905= 1.0514  Log(95% CI) = logOR +/- 1.96* SE = 5.5204, 1.3990  e^5.5204= 249.73  e^1.3990 = 4.05  **aOR = 31.81 (95% CI 4.05 – 249.73)** |
| 1. Cummings 1989 | 1. Have % patients given a follow up appointment, need an effect measure 2. Have biochemically confirmed abstinence >= 9 months, need an effect measure | Intervention 71/466; 40 physicians  Control 21/417; 41 physicians  Average cluster size = (466+417)/81 = 10.90  Design effect for the trial is:  1 +(M-1)ICC = 1 +(10.90-1) x 0.133 = 2.3169  Intervention sample size  466/2.3169= **201**  Intervention cases  71/2.3169= **31**  Control sample size  417/2.3169= **180**  Control cases  21/2.3169= **9**  **Stata code: cci 31 170 9 171**  **OR = 3.46 (95% CI 1.55 – 8.51)**  **Without rounding: OR = 3.39 (95% CI = 1.57 – 7.33 )**  Intervention = 26/1004; 40 physicians  Control = 15/1008; 41 physicians  Average cluster size = (1044 + 1008)/81 = 25.33  Design effect for the trial is:  1 +(M-1)ICC = 1 +(25.33-1) x 0.133 = **4.2463**  Intervention sample size  1004/4.2463= **237**  Intervention cases  26/4.2463= **6**  Control sample size  1008/4.2463= **237**  Control cases  15/4.2463= **4**  **Stata code: cci 6 231 4 233**  **OR = 1.51 (95% CI 0.35 – 7.38)**  **Without rounding: OR = 1.76 (95% CI = 0.47 – 6.52)** |
| 1. Mejia 2013 | We have an adjusted p value, no effect size for   1. Agreed to in-person follow up appointment at 12 months 2. Self-reported tobacco abstinence at 12 months | p= 0.209 and therefore z = 1.2563 = logOR/ SE  OR = (21/729)/ (12/616) = **1.48**  logOR = **0.3911**  SE = 0.3911/1.2563 = **0.3113**  Log(95% CI) = logOR +/- 1.96* SE = 1.0012, -0.2190  e^= 2.72  e^= 0.80  **aOR = 1.48 (95% CI 0.80 – 2.72)**  **Without rounding: OR = 1.48 (95% CI = 0.80 – 2.76 )**  p= 0.435 and therefore z = 0.7807= logOR/ SE  OR = (181/569)/ (144/484) = **1.07**  logOR = **0.0669**  SE = 0.0669/0.7807 = **0.0857**  Log(95% CI) = logOR +/- 1.96* SE = 0.2349, -0.1011  e^0.2349= 1.26  e^-0.1011= 0.90  **aOR = 1.07 (95% CI 0.90 – 1.26)**  **Without rounding: OR = 1.10 (95% CI = 0.86 – 1.40)** |
| 1. Lennox 1998 | Give 2x2 table and ICC as 0.013, need to calculate an effect size | 1. Process outcome (mentioned smoking)   Intervention: 420/529; 8 practices  Control 355/474; 8 practices  Average cluster size = (529 + 474)/16 = 62.6875  Design effect:  1 +(M-1)ICC = 1 +(62.6875-1) x 0.013 = **1.8058**  Intervention sample size  529/1.8058= **293**  Intervention cases  420/1.8058= **233**  Control sample size  474/1.8058= **262**  Control cases  355/1.8058= **197**  **Stata code: cci 233 60 197 65**  **OR = 1.28 (95% CI 0.84 –1.95)**  **Without rounding: OR = 1.29 (95% CI = 0.87 – 1.92 )**   1. Clinical outcome: point prevalence of smoking at 14 months   Intervention: 100/898; 8 practices  Control: 93/795; 8 practices  Average cluster size (898+795)/16 = 105.8125  Design effect for the trial is:  1 +(M-1)ICC = 1 +(105.8125-1) x 0.013 = **1.3626**  Intervention sample size  898/1.3626= **659**  Intervention cases  100/1.3626= **73**  Control sample size  795/1.3626= **583**  Control cases  93/1.3626= **68**  **Stata code: cci 73 586 68 515**  **OR = 0.94 (95% CI 0.65 – 1.36)**  **Without rounding: OR = 0.95 (95% CI = 0.60 – 1.50)** |
| 1. Sinclair 1998 | Design effect was approximately 1. Effect measure calculated from given 2x2 table | Process outcome: pharmacy personnel discussed stopping smoking  Intervention: 113/133  Control: 99/159  Stata code:  cci 113 20 99 60  **OR = 3.42 (95% CI 1.87– 6.41)**  Clinical outcome: 9 month continuous abstinence  Intervention: 26/217  Control: 19/257  Stata code:  cci 26 191 19 238  **OR = 1.71 (95% CI 0.88– 3.36)** |
| 1. Kottke 1989 | Have 2x2 table and results are given at the level of the clinician | Using t test of proportions, can get a p value of 0.0249 (paper states ‘NS but we are including 2/3 arms workshop and control)  Intervention: 10.3/100  Control 3.8/100  Stata code: cci 103 897 38 962  OR = 2.90  p= 0.0249 and therefore z = 2.2429 = logOR/ SE  logOR = 1.0671  SE = 1.0671/2.2429 = 0.4758  Log(95% CI) = logOR +/- 1.96* SE = 1.9997. 0.1345  e^1.9997= 7.386  e^0.1345= 1.144  **aOR = 2.90 (95% CI 1.14 – 7.39)** |
| 1. Olano Espinosa 2013 | Have 2x2 table but not adjusted for clustering. Also ‘0’ in one cell | Use the peto method to manage ‘0’  Stata code:  set obs 1  generate var1 = 31 in 1  generate var2 = 2718 in 1  generate var3 = 0 in 1  generate var4 = 3192 in 1  list  gen nonevents1=var2-var1  gen nonevents2=var4-var3  list  metan var1 nonevents1 var3 nonevents2, peto  **OR = 8.90**  Then adjust for clustering using imputed ICC from other studies:  Adjusting CI for clustering:  Design effect for the trial is:  (2718 +3192)/35 HCCs = 168.86  1 +(M-1)ICC = 1 +(168.86-1) x 0.133 = 23.325  SQRT(23.325) = 4.830  display 2.185-1.477  display 2.894-2.185  scalar halfwidth = 2.185-1.477  display halfwidth  display 2.185-halfwidth*4.83  display 2.185+halfwidth*4.83  display exp(2.185-halfwidth*4.83)  display exp(2.185+halfwidth*4.83)  **Therefore aOR = 8.90 (95% CI 0.29 – 271.68)** |
| 1. Moore 2003 | Have difference in weight between intervention and control arms and SD at baseline | Rearranging Chinn 2000 formula and using the SD of the control group weight (17.4kg)at baseline  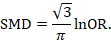  Effect size = EXP((3.1415*(1.0**/17.4**)/(SQRT(3))) = 1.11  Lower CI = EXP((3.1415*(-1.9**/17.4**)/(SQRT(3))) = 0.82  Upper CI = EXP((3.1415*(3.9**/17.4**)/(SQRT(3))) = 1.50 |
| 1. Baldeon 2018 | Say adjusted and unadjusted analysis were similar and so only report the unadjusted analysis. Decided that as give no further detail e.g., an ICC we will impute an ICC and adjust for clustering | Process outcome (referral to nutrition counselling):  Intervention: 11/111; 3 health care centres  Control: 11/81; 3 health care centres  Average cluster size = (111+81)/6 = 32  Design effect for the trial is:  1 +(M-1)ICC = 1 +(32-1) x 0.133 = 5.123  Intervention sample size  111/5.123= **22**  Intervention cases  11/5.123= **2**  Control sample size  81/5.123= **16**  Control cases  11/5.123= **2**  **Stata code: cci 2 20 2 14**  **OR = 0.70 (95% CI 0.05 – 10.84)**  **Without rounding: OR = 0.70 (95% CI = 0.09 – 5.25 )** |
| 1. Babor 2004 | Give % in each group and total n of patients | Patients, n=1329  As 10 practices intervention and 5 practices control, assume that patients distributed in a ratio 10:5  Therefore:  Patients intervention = 886 – 47% asked about alcohol consumption = 416  Patients control = 443 – 21.9% asked about alcohol consumption = 97  Average cluster size = 1329/15 = 88.6  Design effect for the trial is:  1 +(M-1)ICC = 1 +(88.6-1) x 0.133 = 12.65  Intervention sample size  886/12.65= **70**  Intervention cases  416/12.65= **33**  Control sample size  443/12.65= **35**  Control cases  97/12.65= **8**  **Stata code: cci 33 37 8 27**  **OR = 3.01 (95% CI 1.12 – 8.68)**  **Without rounding: OR = 3.16 (95% CI = 1.25 – 7.99 )** |
| **Financial Incentives** | | |
| 1. Roski 2003 | Have 2x2 table (combining incentive and registry arm) and adjusted p value. Need to calculate and effect size for both process and clinical outcomes | 1. Process outcome (smokers who were offered assistance to quit at last visit between baseline and follow up)   Combining arms:  Intervention = (0.314*352) + (0.367*182) = **177** events  Therefore, intervention: **177/534**  Control: **112/329**  p = 0.451 and therefore z = 0.7537= logOR/ SE  OR = (177/357)/ (112/217) = **0.96**  logOR = -0.0402  SE = -0.0402 /0.7537= -0.0533  Log(95% CI) = logOR +/- 1.96* SE = 0.0642, -0.1447  e^-1.0848 = 0.87  e^1.0045= 1.07  **aOR = 0.96 (95% CI 0.87 – 1.07)**   1. Clinical outcomes (Current non-smoker -7-day sustained abstinence from smoking at 6 months)   Combining arms:  Intervention = (0.224* 229) + 0.217*139) = **81 events**  Therefore, intervention: **81/ 368**  Control = **39/205**  p= 0.20 and therefore z = 1.2816= logOR/ SE  OR = (81/287)/ (39/166) = **1.20**  logOR = 0.1834  SE = 0.1834 /1.2816= **0.1431**  Log(95% CI) = logOR +/- 1.96* SE =0.4639, -0.0971  e^0.4639= 1.59  e^-0.0971 = 0.91  **aOR = 1.20 (95% CI 0.91- 1.59)** |
|  |  |  |
| **Electronic patient registry** | | |
| 1. Fiore 2019 | Have 2x2 table, but do not adjust for clustering. | Health system A  Average cluster size: (From supplementary material, no. of current smokers) 3020 + 3415 = 6435  6435/11 clinics = 585  Design effect for the trial is:  1 +(M-1)ICC = 1 +(585-1) x 0.133 = 78.672  Intervention sample size  3415/77.27= **44**  Intervention cases  610/77.27= **8**  Control sample size  3020/77.27= **38**  Control cases  115/77.27= **1**  **Stata code: cci 8 36 1 37**  **aOR = 8.22 (95% CI 1.00 – 374.43)**  **Without rounding: OR = 5.49 (95% CI = 0.88 – 34.12 )**  Health system B  Average cluster size (from supplementary material, no. of current smokers): 4135 + 4066 = 8201  8201/12 clinics = 683.42  Design effect for the trial is:  1 +(M-1)ICC = 1 +(683.42-1) x 0.133 = 91.76  Intervention sample size  4066/91.76= **44**  Intervention cases  770/91.76= **8**  Control sample size  4135/91.76= **45**  Control cases  216/91.76= **2**  **Stata code: cci 8 36 2 43**  **aOR = 4.78 (95% CI 0.86 – 48.19)**  **Without rounding: OR = 4.24 (95% CI = 0.93 – 19.22 )** |
| 1. Boston 2023 | Need to combine CDS groups and then adjust for clustering | Process level outcome  Any tobacco cessation Rx recommendation  9.1% of 3006 = 274  6.9% of 7110 = 491  6.7% of 9543 = 639  Combining groups CDS+ and CDS- therefore = (274 + 491)/ (3006+7110) = 765/ 10116; 38 clinics  Control = 639/9543; 27 clinics  Average cluster size = (10116 + 9543)/ (38+27) = 302.446  Design effect for the trial is:  1 +(M-1)ICC = 1 +(302.446-1) x 0.133 = 41.094  Intervention sample size  10116/41.094= **246**  Intervention cases  765/41.094= **19**  Control sample size  9543/41.094= **232**  Control cases  639/41.094= **16**  **Stata code: cci 19 227 16 216**  **OR = 1.13 (95% CI 0.53 – 2.41)**  **Without rounding: OR = 1.14 (95% CI = 0.57 – 2.29)** |
| 1. Minian 2002 | Have change in exercise, need to convert to an OR | ‘Mean exercise minutes changed from 32 (SD 44.7) to 110 (SD 196.1) in the intervention arm and from 32 (SD 45.1) to 113 (SD 195.1) in the control arm (group effect: B=-3.7 minutes; 95% CI -17.8 to 10.4; P=.61)’  SD at baseline in control group = 45.1 minutes  Chinn 2000 formula:  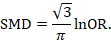  Effect size = EXP((3.1415*(-3.7/45.1)/(SQRT(3))) = **0.86**  Lower CI = EXP((3.1415*-17.8/45.1)/(SQRT(3))) = **0.49**  Upper CI = EXP((3.1415*10.4/45.1)/(SQRT(3))) = **1.52** |
