# Supplementary material for: Strategies to improve the implementation of preventive care in primary care: a systematic review and meta-analysis
Source: BMC Med. 2024 Sep 27;22:412. doi: 10.1186/s12916-024-03588-5 (PMC11437661; doi:10.1186/s12916-024-03588-5)
Supplement: Supplementary file 3 — Additional file 3: Figures S1-S7. FigS1 – Meta-analysis summary diamonds as random effects (main analysis), Hartung-Knapp-Sidik-Jonkman (HKSJ) and inverse variance heterogeneity (IVhet) models for process outcomes. FigS2 - Meta-analysis summary diamonds as random effects (main analysis), Hartung-Knapp-Sidik-Jonkman (HKSJ) and inverse variance heterogeneity model (IVhet) models for behavioural outcomes. FigS3 - Random effects meta-analysis summary diamonds excluding studies at high risk of bias and those that required data imputation for process outcomes. FigS4 - Random effects meta-analysis summary diamonds excluding studies at high risk of bias and those that required data imputation for behavioural outcomes. FigS5 - Random effects meta-analysis of behavioural outcomes, excluding Moore 2003, Welzel 2021 and Goodfellow 2016. FigS6 - Random effects meta-analysis summary diamonds of health behaviour subgroups for process outcomes. FigS7 - Random effects meta-analysis summary diamonds of health behaviour subgroups for behavioural outcomes. [file 12916_2024_3588_MOESM3_ESM.docx]

**Figure S1: Meta-analysis summary diamonds as random effects (main analysis), Hartung-Knapp-Sidik-Jonkman (HKSJ) and inverse variance heterogeneity (IVhet) models for process outcomes**

**Figure S2: Meta-analysis summary diamonds as random effects (main analysis), Hartung-Knapp-Sidik-Jonkman (HKSJ) and inverse variance heterogeneity model (IVhet) models for behavioural outcomes**

**Figure S3: Random effects meta-analysis summary diamonds excluding studies at high risk of bias and those that required data imputation for process outcomes**

**Figure S4: Random effects meta-analysis summary diamonds excluding studies at high risk of bias and those that required data imputation for behavioural outcomes.**

**Figure S5: Random effects meta-analysis of behavioural outcomes, excluding Moore 2003, Welzel 2021 and Goodfellow 2016**

**
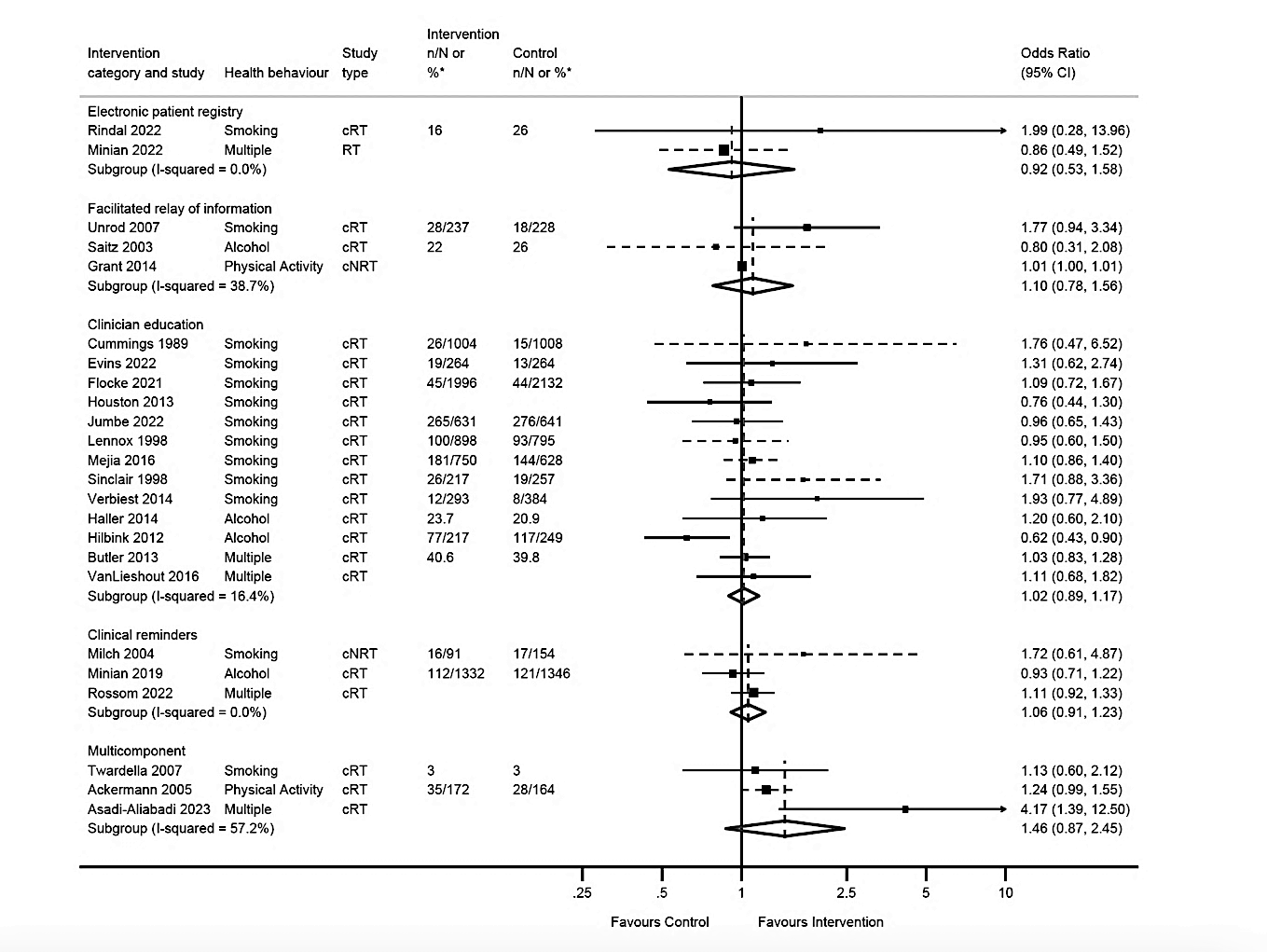
**

**Figure S6: Random effects meta-analysis summary diamonds of health behaviour subgroups for process outcomes**

**Figure S7: Random effects meta-analysis summary diamonds of health behaviour subgroups for behavioural outcomes**
