# Supplementary material for: Strategies to improve the implementation of preventive care in primary care: a systematic review and meta-analysis
Source: BMC Med. 2024 Sep 27;22:412. doi: 10.1186/s12916-024-03588-5 (PMC11437661; doi:10.1186/s12916-024-03588-5)
Supplement: Supplementary file 4 — Additional file 4: Stata code. [file 12916_2024_3588_MOESM4_ESM.docx]

******* Code for meta-analysis and forest plot for process outcomes********

update all

clear all

set maxvar 10000

set more off

import excel "/Users/lauraheath/Library/Mobile Documents/com~apple~CloudDocs/Documents/DPhil/1. Systematic Review/Systematic Review/Code for meta-analysis/Master/Overall_forestplots.xlsx", sheet("Process") firstrow

ssc install admetan

gen logeffect=log(em)

gen loglower=log(ci_l)

gen logupper=log(ci_u)

** Model 1: standard random effects **

noisily admetan logeffect loglower logupper, by(Interventioncategory) nowt eform lcols(Study Healthbehaviour Studytype InterventionnN ControlnN) effect(Odds Ratio) random nooverall nowarning forestplot(favours(Favours Control # Favours Intervention) xlabel(0.25 0.5 1 2.5 5 10 20 30 40 50, force) astext(60) leftjustify classic boxscale(12) plotid(impute) graphregion(color(white)) ci1opts(lwidth(vthin) msize(vsmall)) ci2opts(lpattern(vshortdash) lwidth(vthin) msize(vsmall)))

** Model 2: hksj **

admetan logeffect loglower logupper, by(Interventioncategory) nowt eform lcols(Study HealthBehaviour StudyType InterventionnN ControlnN) effect(Odds Ratio) model(hksj) nooverall forestplot(favours(Favours Control # Favours Intervention) xlabel(0.5 1 2.5 5 10 20 30) leftjustify)

** Model 3: ivhet **

admetan logeffect loglower logupper, by(Interventioncategory) nowt eform lcols(Study HealthBehaviour StudyType InterventionnN ControlnN) effect(Odds Ratio) model(ivhet) nooverall forestplot(favours(Favours Control # Favours Intervention) xlabel(0.5 1 2.5 5 10 20 30) leftjustify)

** Model 4: standard random effects senstivity analysis - remove those at high risk of bias **

noisily admetan logeffect loglower logupper if sensitivity==1, by(Interventioncategory) nowt eform lcols(Study Healthbehaviour Studytype InterventionnN ControlnN) effect(Odds Ratio) random nooverall forestplot(favours(Favours Control # Favours Intervention) xlabel(0.25 0.5 1 2.5 5 10 20 30 40 50, force) astext(60) leftjustify classic boxscale(12) plotid(impute) ci1opts(lwidth(vthin) msize(vsmall)) ci2opts(lpattern(vshortdash) lwidth(vthin) msize(vsmall)))

** Model 5: standard random effects sensitivity analysis - remove those with imputed data

noisily admetan logeffect loglower logupper if impute==1, by(Interventioncategory) nowt eform lcols(Study Healthbehaviour Studytype InterventionnN ControlnN) effect(Odds Ratio) random nooverall forestplot(favours(Favours Control # Favours Intervention) xlabel(0.25 0.5 1 2.5 5 10 20 30 40 50, force) astext(60) leftjustify classic boxscale(12) plotid(impute) ci1opts(lwidth(vthin) msize(vsmall)) ci2opts(lpattern(vshortdash) lwidth(vthin) msize(vsmall)))

** Model 6: standard random effects subgroup analysis - smoking

noisily admetan logeffect loglower logupper if smoking==1, by(Interventioncategory) nowt eform lcols(Study Healthbehaviour Studytype InterventionnN ControlnN) effect(Odds Ratio) random nooverall forestplot(favours(Favours Control # Favours Intervention) xlabel(0.25 0.5 1 2.5 5 10 20 30 40 50, force) astext(60) leftjustify classic boxscale(12) plotid(impute) ci1opts(lwidth(vthin) msize(vsmall)) ci2opts(lpattern(vshortdash) lwidth(vthin) msize(vsmall)))

** Model 6: standard random effects subgroup analysis - alcohol

noisily admetan logeffect loglower logupper if alcohol==1, by(Interventioncategory) nowt eform lcols(Study Healthbehaviour Studytype InterventionnN ControlnN) effect(Odds Ratio) random nooverall forestplot(favours(Favours Control # Favours Intervention) xlabel(0.25 0.5 1 2.5 5 10 20 30 40 50, force) astext(60) leftjustify classic boxscale(12) plotid(impute) ci1opts(lwidth(vthin) msize(vsmall)) ci2opts(lpattern(vshortdash) lwidth(vthin) msize(vsmall)))

** Model 6: standard random effects subgroup analysis - obesity

noisily admetan logeffect loglower logupper if obesity==1, by(Interventioncategory) nowt eform lcols(Study Healthbehaviour Studytype InterventionnN ControlnN) effect(Odds Ratio) random nooverall forestplot(favours(Favours Control # Favours Intervention) xlabel(0.25 0.5 1 2.5 5 10 20 30 40 50, force) astext(60) leftjustify classic boxscale(12) plotid(impute) ci1opts(lwidth(vthin) msize(vsmall)) ci2opts(lpattern(vshortdash) lwidth(vthin) msize(vsmall)))

** Model 6: standard random effects subgroup analysis - multiple

noisily admetan logeffect loglower logupper if multiple==1, by(Interventioncategory) nowt eform lcols(Study Healthbehaviour Studytype InterventionnN ControlnN) effect(Odds Ratio) random nooverall forestplot(favours(Favours Control # Favours Intervention) xlabel(0.25 0.5 1 2.5 5 10 20 30 40 50, force) astext(60) leftjustify classic boxscale(12) plotid(impute) ci1opts(lwidth(vthin) msize(vsmall)) ci2opts(lpattern(vshortdash) lwidth(vthin) msize(vsmall)))

*** Sensitivity Analysis 1 - different meta-analysis techniques (summary)

clear

import excel "/Users/lauraheath/Library/Mobile Documents/com~apple~CloudDocs/Documents/DPhil/1. Systematic Review/Systematic Review/Code for meta-analysis/Master/Sensitivity_analysis.xlsx", sheet("Process") firstrow

gen order=_n

gen beta=log(or)

gen lbeta=log(lower)

gen ubeta=log(upper)

label define groupingL 1 "Clinician reminders: Main analysis" 2 "HKSJ" 3 "IVHet" ///

4 "Clinician education: Main analysis" 5 "HKSJ" 6 "IVHet" ///

7 "Electronic patient registry: Main analysis" 8 "HKSJ" 9 "IVHet" ///

10 "Facilitated relay of information: Main analysis" 11 "HKSJ" 12 "IVHet" ///

13 "Financial incentives: Main analysis" 14 "HKSJ" 15 "IVHet" ///

16 "Multicomponent: Main analysis" 17 "HKSJ" 18 "IVHet" ///

19 "Team changes: Main analysis" 20 "HKSJ" 21 "IVHet"

label values order groupingL

label variable order "Intervention group and analysis"

decode order, gen(label2)

gen _USE=3

forestplot beta lbeta ubeta, or nooverall by(order) nohet nowt nokeepvars labels(label2) xlabel(0.1 1 10 100) range(0.1 100) leftjustify graphregion(color(white))

*** Sensitivity Analysis 2 excluding high RoB or imputation

clear

import excel "/Users/lauraheath/Library/Mobile Documents/com~apple~CloudDocs/Documents/DPhil/1. Systematic Review/Systematic Review/Code for meta-analysis/Master/Sensitivity_analysis.xlsx", sheet("Process - Sens") firstrow

gen order=_n

gen beta=log(or)

gen lbeta=log(lower)

gen ubeta=log(upper)

label define groupingL 1 "Clinician reminders: Main analysis" 2 "Exclude high RoB" 3 "Exclude imputation" ///

4 "Clinician education: Main analysis" 5 "Exclude high RoB" 6 "Exclude imputation" ///

7 "Electronic patient registry: Main analysis" 8 "Exclude high RoB" 9 "Exclude imputation" ///

10 "Facilitated relay of information: Main analysis" 11 "Exclude imputation" ///

12 "Financial incentives: Main analysis" 13 "Exclude high RoB" ///

14 "Multicomponent: Main analysis" 15 "Exclude high RoB" 16 "Exclude imputation"

label values order groupingL

label variable order "Intervention group and analysis"

decode order, gen(label2)

gen _USE=3

*** Fix imported missing data rows

drop if (or==. & lower==. & upper==.)

forestplot beta lbeta ubeta, or nooverall by(order) nohet nowt nokeepvars labels(label2) xlabel(0.1 1 10) range(0.1 100) leftjustify graphregion(color(white))

*** Subgroup analysis

clear

import excel "/Users/lauraheath/Library/Mobile Documents/com~apple~CloudDocs/Documents/DPhil/1. Systematic Review/Systematic Review/Code for meta-analysis/Master/Sensitivity_analysis.xlsx", sheet("Process - Sub") firstrow

gen order=_n

gen beta=log(or)

gen lbeta=log(lower)

gen ubeta=log(upper)

label define groupingL 1 "Clinician reminders: Main analysis" 2 "Smoking" ///

3 "Clinician education: Main analysis" 4 "Smoking" 5 "Alcohol" 6 "Obesity" 7 "Multiple" ///

8 "Electronic patient registry: Main analysis" 9 "Smoking" ///

10 "Facilitated relay of information: Main analysis" 11 "Smoking" ///

12 "Multicomponent: Main analysis" 13 "Smoking" 14 "Alcohol" 15 "Multiple"

label values order groupingL

label variable order "Intervention group and analysis"

decode order, gen(label2)

gen _USE=3

*** Fix imported missing data rows

drop if (or==. & lower==. & upper==.)

forestplot beta lbeta ubeta, or nooverall by(order) nohet nowt nokeepvars labels(label2) xlabel(0.1 1 10) range(0.1 100) leftjustify graphregion(color(white))

******* Code for meta-analysis and forest plots of clinical outcomes ********

update all

clear all

set maxvar 10000

set more off

import excel "/Users/lauraheath/Library/Mobile Documents/com~apple~CloudDocs/Documents/DPhil/1. Systematic Review/Systematic Review/Code for meta-analysis/Master/Overall_forestplots.xlsx", sheet("Outcome") firstrow

ssc install admetan

gen logeffect=log(em)

gen loglower=log(ci_l)

gen logupper=log(ci_u)

** Model 1: standard random effects **

admetan logeffect loglower logupper, by(Interventioncategory) nowt eform lcols(study Healthbehaviour Studytype InterventionnN ControlnN) effect(Odds Ratio) random nooverall nowarning forestplot(favours(Favours Control # Favours Intervention) xlabel(0.25 0.5 1 2.5 5 10, force) astext(60) leftjustify classic boxscale(20) plotid(impute) graphregion(color(white)) ci1opts(lwidth(vthin) msize(vsmall)) ci2opts(lpattern(shortdash) lwidth(vthin) msize(vsmall)))

** Model 2: hksj **

admetan logeffect loglower logupper, by(Interventioncategory) nowt eform lcols(Study HealthBehaviour StudyType InterventionnN ControlnN) effect(Odds Ratio) model(hksj) nooverall forestplot(favours(Favours Control # Favours Intervention) xlabel(0.5 1 2.5 5 10 20 30) leftjustify)

** Model 3: ivhet **

admetan logeffect loglower logupper, by(Interventioncategory) nowt eform lcols(Study HealthBehaviour StudyType InterventionnN ControlnN) effect(Odds Ratio) model(ivhet) nooverall forestplot(favours(Favours Control # Favours Intervention) xlabel(0.5 1 2.5 5 10 20 30) leftjustify)

** Model 4: standard random effects sensitivity analysis - remove those at high risk of bias **

noisily admetan logeffect loglower logupper if sensitivity==1, by(Interventioncategory) nowt eform lcols(Study Healthbehaviour Studytype InterventionnN ControlnN) effect(Odds Ratio) random nooverall forestplot(favours(Favours Control # Favours Intervention) xlabel(0.25 0.5 1 2.5 5 10 20 30 40 50, force) astext(60) leftjustify classic boxscale(12) plotid(impute) ci1opts(lwidth(vthin) msize(vsmall)) ci2opts(lpattern(vshortdash) lwidth(vthin) msize(vsmall)))

** Model 5: standard random effects sensitivity analysis - remove those with imputed data

noisily admetan logeffect loglower logupper if impute==1, by(Interventioncategory) nowt eform lcols(Study Healthbehaviour Studytype InterventionnN ControlnN) effect(Odds Ratio) random nooverall forestplot(favours(Favours Control # Favours Intervention) xlabel(0.25 0.5 1 2.5 5 10 20 30 40 50, force) astext(60) leftjustify classic boxscale(12) plotid(impute) ci1opts(lwidth(vthin) msize(vsmall)) ci2opts(lpattern(vshortdash) lwidth(vthin) msize(vsmall)))

** Model 6: standard random effects subgroup analysis - smoking

noisily admetan logeffect loglower logupper if smoking==1, by(Interventioncategory) nowt eform lcols(Study Healthbehaviour Studytype InterventionnN ControlnN) effect(Odds Ratio) random nooverall forestplot(favours(Favours Control # Favours Intervention) xlabel(0.25 0.5 1 2.5 5 10 20 30 40 50, force) astext(60) leftjustify classic boxscale(12) plotid(impute) ci1opts(lwidth(vthin) msize(vsmall)) ci2opts(lpattern(vshortdash) lwidth(vthin) msize(vsmall)))

** Model 6: standard random effects subgroup analysis - alcohol

noisily admetan logeffect loglower logupper if alcohol==1, by(Interventioncategory) nowt eform lcols(Study Healthbehaviour Studytype InterventionnN ControlnN) effect(Odds Ratio) random nooverall forestplot(favours(Favours Control # Favours Intervention) xlabel(0.25 0.5 1 2.5 5 10 20 30 40 50, force) astext(60) leftjustify classic boxscale(12) plotid(impute) ci1opts(lwidth(vthin) msize(vsmall)) ci2opts(lpattern(vshortdash) lwidth(vthin) msize(vsmall)))

** Model 6: standard random effects subgroup analysis - obesity

noisily admetan logeffect loglower logupper if obesity==1, by(Interventioncategory) nowt eform lcols(Study Healthbehaviour Studytype InterventionnN ControlnN) effect(Odds Ratio) random nooverall forestplot(favours(Favours Control # Favours Intervention) xlabel(0.25 0.5 1 2.5 5 10 20 30 40 50, force) astext(60) leftjustify classic boxscale(12) plotid(impute) ci1opts(lwidth(vthin) msize(vsmall)) ci2opts(lpattern(vshortdash) lwidth(vthin) msize(vsmall)))

** Model 6: standard random effects subgroup analysis - multiple

noisily admetan logeffect loglower logupper if multiple==1, by(Interventioncategory) nowt eform lcols(Study Healthbehaviour Studytype InterventionnN ControlnN) effect(Odds Ratio) random nooverall forestplot(favours(Favours Control # Favours Intervention) xlabel(0.25 0.5 1 2.5 5 10 20 30 40 50, force) astext(60) leftjustify classic boxscale(12) plotid(impute) ci1opts(lwidth(vthin) msize(vsmall)) ci2opts(lpattern(vshortdash) lwidth(vthin) msize(vsmall)))

*** Sensitivity Analysis 1 - different meta-analysis techniques (summary)

clear

import excel "/Users/lauraheath/Library/Mobile Documents/com~apple~CloudDocs/Documents/DPhil/1. Systematic Review/Systematic Review/Code for meta-analysis/Master/Sensitivity_analysis.xlsx", sheet("Outcome") firstrow

gen order=_n

gen beta=log(or)

gen lbeta=log(lower)

gen ubeta=log(upper)

label define groupingL 1 "Clinical reminders: Main analysis" 2 "HKSJ" 3 "IVHet" ///

4 "Clinician education: Main analysis" 5 "HKSJ" 6 "IVHet" ///

7 "Electronic patient registry: Main analysis" 8 "HKSJ" 9 "IVHet" ///

10 "Facilitated relay of information: Main analysis" 11 "HKSJ" 12 "IVHet" ///

13 "Multicomponent: Main analysis" 14 "HKSJ" 15 "IVHet"

label values order groupingL

label variable order "Intervention group and analysis"

decode order, gen(label2)

gen _USE=3

forestplot beta lbeta ubeta, or nooverall by(order) nohet nowt nokeepvars labels(label2) xlabel(0.1 1 10) range(0.1 50) leftjustify graphregion(color(white))

*** Sensitivity Analysis - excluding high RoB or imputation

clear

import excel "/Users/lauraheath/Library/Mobile Documents/com~apple~CloudDocs/Documents/DPhil/1. Systematic Review/Systematic Review/Code for meta-analysis/Master/Sensitivity_analysis.xlsx", sheet("Outcome - Sens") firstrow

gen order=_n

gen beta=log(or)

gen lbeta=log(lower)

gen ubeta=log(upper)

label define groupingL 1 "Clinical reminders: Main analysis" 2 "Exclude imputation" ///

3 "Clinician education: Main analysis" 4 "Exclude high RoB" 5 "Exclude imputation" ///

6 "Multicomponent: Main analysis" 7 "Exclude high RoB" 8 "Exclude imputation"

label values order groupingL

label variable order "Intervention group and analysis"

decode order, gen(label2)

gen _USE=3

*** Fix imported missing data rows

drop if (or==. & lower==. & upper==.)

forestplot beta lbeta ubeta, or nooverall by(order) nohet nowt nokeepvars labels(label2) xlabel(0.1 1 10) range(0.1 50) leftjustify graphregion(color(white))

*** Subgroup analysis

clear

import excel "/Users/lauraheath/Library/Mobile Documents/com~apple~CloudDocs/Documents/DPhil/1. Systematic Review/Systematic Review/Code for meta-analysis/Master/Sensitivity_analysis.xlsx", sheet("Outcome - Sub") firstrow

gen order=_n

gen beta=log(or)

gen lbeta=log(lower)

gen ubeta=log(upper)

label define groupingL 1 "Clinician education: Main analysis" 2 "Smoking" 3 "Alcohol" ///

4 "Obesity" 5 "Multiple"

label values order groupingL

label variable order "Intervention group and analysis"

decode order, gen(label2)

gen _USE=3

*** Fix imported missing data rows

drop if (or==. & lower==. & upper==.)

forestplot beta lbeta ubeta, or nooverall by(order) nohet nowt nokeepvars labels(label2) xlabel(0.1 1 10) range(0.1 50) leftjustify graphregion(color(white))
