# Supplementary material for: Strategies to improve the implementation of preventive care in primary care: a systematic review and meta-analysis
Source: BMC Med. 2024 Sep 27;22:412. doi: 10.1186/s12916-024-03588-5 (PMC11437661; doi:10.1186/s12916-024-03588-5)
Supplement: Supplementary file 5 — Additional file 5: Table S1 - Characteristics of included studies. [file 12916_2024_3588_MOESM5_ESM.docx]

**Additional Table S1: Characteristics of included studies.**

| Study, year | Study design | Location | Health behaviour | N randomised | Main inclusion criteria | Main exclusion criteria | % male | Average age | Ethnicity | Intervention description | Comparator | Follow- up |
| --- | --- | --- | --- | --- | --- | --- | --- | --- | --- | --- | --- | --- |
| **Clinical Reminders** | | | | | | | | | | | | |
| Baer 2016 | cRT | MA, USA | Obesity | 12 clinics | All patients with at least 1 visit between June 11, 2012, and December 10, 2012, BMI ≥25mg/kg2 | Providers with <50 patients during this period. | Patient: 35.3% male | Patient: 50 years | 15.5% Hispanic/ Latino | Reminders and alerts for overweight/ obesity | Usual care | 12 months |
| Banerjee 2013 | RT | PA, USA | Obesity | 843 | Patient with obesity, obesity not on their problem list, had not had obesity addressed, not pregnant, had an appointment within 5 months of the follow up | Not meeting inclusion criteria, patients of 3 providers at the study site who were aware of the study | Patient: 27% male | Patient: 47 years | Patient: 80% white | Obesity prompt | Usual care | 5 months |
| Dubey 2006 | cRT | Toronto, Canada | Multiple | 4 clinics | Physicians consented to a trial on prevention | Did not meet inclusion criteria | Patient: 39.5% male; Physician: 89% male | Patient: 44.4 years; Physician: 42.0 years | Not given | Prevention checklist | Usual care | 5 months |
| Linder 2009 | cRT | MA, USA | Smoking | 26 clinics | All 26 affiliated clinics, patients who made a consultation 9 months before and patients who made a consultation in the study period | Not reported | Physicians: 46% male; Smokers: 39% male | Smokers: 48.5 years | Smokers: 64.4% white | Smoking cessation reminders | Usual care | 9 months |
| Milch 2004 | cNRT | Boston, MA, USA | Smoking | 5 teams, 38 HCPs | All patients attending the practice between April - September 1999, current smokers | Not regular and current smokers, denied on follow-up interview that they were smokers, denied to their clinician they smoked or were terminally ill, patients seen by the chief of practice (who allocated intervention/control) | Patient: 49% male | Patient: 41 years | 72% white in patient population | Vital signs stamp | Usual care | 9 months |
| Minian 2019 | cRT | Ontario, Canada | Alcohol | 221 clinics | Enrolled in the STOP smoking cessation programme | Not using the online portal e.g., paper-based referrals | Patient: 55% male | Patient age: 47.9 years | Not given | Clinical decision support prompt | Usual care (no prompts) | 6 months |
| Rossom 2022 | cRT | Midwest, USA | Multiple | 80 clinics | PCPs must be a general internist, family physician, or adult-care non-obstetric nurse practitioner. Provide ongoing primary care for adults with SMI in 2012 and provide written informed consent. Patients must meet the study criteria to be diagnosed with SMI (one inpatient or two outpatient ICD-9 codes for schizophrenia, schizoaffective disorder, or bipolar disorder; >17 and <80 years old; not pregnant; Charlson comorbidity score 3 or less; be linked to a consented PCP; have at least 1 primary care visit with a consented PCP in the 12m prior to clinic randomisation | Not meeting inclusion criteria, patients in nursing homes or hospices or those with active cancer care | Patients: 44.9% men | Patients: 48.4 years | Patients: 83.7% white | Electronic Health Record Best Practice advisories to print information of modifiable CV risk factors | Usual care | 12 months |
| Rothemich 2008 | cRT | Virginia, USA | Smoking | 18 practices, 73 clinicians | Practices with at least 2 clinicians who specialised in adult primary care | Residency programs, clinics serving special populations, practices not under institutional review board, practices with an existing systematic tobacco identification and reminder system. | Physicians: 69% male Patients: 60% male | Physicians: not given; Patients: 52 years | Physicians: 84% white | Vital sign stamp | Usual care | 6 months |
| Wadlin 2021 | cRT | Maryland, USA | Smoking | 15 clinics | All primary care offices included if had visits from adult >= 18 years. Encounters were eligible if the patient was labelled as currently smoking tobacco | Other encounters where the BPA fired e.g., nursing or medication refill were excluded | Not given | Not given | Not given | Best practice alerts | Usual care | 6 months |
| **Clinician Education** | | | | | | | | | | | | |
| Adams 1998 | cRT | MA, USA | Alcohol | 29 healthcare providers; 530 patients | High risk drinkers were included | Not described | Providers 41% male; patients 63% male | Providers: 37.8 years (SD 5.4); patients 45.1 years (SD 13.9) | Not given | Alcohol intervention training used a 'patient-centred' counselling approach that elicits active patient involvement in behaviour change | Usual care | 6-32 months, average 19 months |
| Anderson 2021 | cRT | Bogata (Columbia)Mexico City (Mexico) and lima (Peru) | Alcohol | 29 PHCC (use hypothesis 2) | Fully qualified HCP, written consent | Not described | Providers 23.1% male | Providers: 38.6 years | Not given | 1.5-2 hours of tailored training | Usual care | 4.75 months |
| Babor 2004 | cRT | USA | Alcohol | 173 healthcare professionals | Each managed care organisation was required to have at least three comparably sized Family or Internal Medicine clinics with annual visits of at least 7,000 unduplicated adult patients, a designated MCO liaison to coordinate SBI activities, and no current alcohol screening programs. | Not described | Healthcare professionals (inc. medical students): 40% male | Healthcare professionals (inc. medical students): 35 years | not given | Cutting back training curriculum | Usual Care | 3 months |
| Baldeon 2018 | cRT | Ecuador | Obesity | 6 health care systems | Age ≥ 30 years and BMI ≥ 25 | Patients taking lipid-lowering medications, those with known coronary heart disease, and those with secondary causes of hyperlipidaemia were excluded. Also, those who declined to participate, did not come to the appointment, were not accessible, or did not meet the definitive eligibility criteria | Patients 30% men | Patients similar in age (value not given) | Not given | Training: didactic component on the relationship between behaviour and CVD risk; training resources; patient-centred counselling; clinical guidelines | Usual care | 6 months |
| Butler 2013 | cRT | Wales | Multiple | 53 general practitioners | Patients Alcohol—Score >4 for men or >3 for women on AUDIT-C Diet—Consumption of <5 fruit and vegetable items per day or ≥3 servings of any of the subset of DINE fat items Exercise—<30 minutes for at least 5 days a week of vigorous or moderate exercise Smoking—≥1 cigarettes smoked per day. Aged 18 or older; able to provide informed consent | If considered inappropriate by the clinician; unable to complete the questionnaire in English | GPs: 34%; patients 38% | GPs: not given; patients: 50.9 (SD 49.55) | not given | Behaviour change counselling was developed from motivational interviewing. Clinicians were trained using a blended learning programme called Talking Lifestyles | Usual care | 12 months |
| Campbell-Scherer 2019 | cRT | Alberta, Canada | Obesity | 24 clinics | All allied healthcare professionals and adult patient visits were included in the study | Clinics were excluded for lack of team or predominantly telephone-based visits | Not given | Nurses: 46.8 years | Not given | 12 x 2-hour interactive educational sessions | Usual care | 9 months |
| Corelli 2022 | cRT | California USA | Smoking | 63 technicians | (a) a private counselling area, (b) the ability to relocate the non-prescription nicotine replacement therapy (NRT) medications from the customer service area at the front of the store to the pharmacy area, and (c) no planned construction in the 12-week study | Stores in Los Angeles County because of a concurrent tobacco cessation program | Technicians: 22% male | Technicians: 38.6 years | Technicians: 58% white | Live training session in addition to self-study training module | Self-study training module | 12 weeks |
| Cummings 1989 | cRT | California, USA | Smoking | 81 internists | All smokers who made a visit to any doctor participating in the study were included | Excluded physicians who had fewer than 20 patient visits a week, patients who did not speak English | Physicians:71.5% male; Patients: 45% male | Physician: not given; Patients: 45.5 years | Not given | 3 x 1-hour seminars to demonstrate and practice counselling about smoking cessation | Self-help booklet | 1 year |
| Evins 2022 | cRT | USA | Smoking | 77 clinics | All adults who smoked tobacco and were eligible for Department of Mental Health psychiatric rehabilitation services for serious mental illness through the largest two behavioural health organisations | Declined to participate, not outpatients, received primary care at a clinic serving fewer than 3 participants | 70% male | 47.8 years (patients) | 45.5% white | Physician education only arm - educational outreach to primary care staff | Usual care | 2 years |
| Flocke 2021 | cRT | Cleveland, Ohio, USA | Smoking | 8 sites | Not given | Not given | Patients: 37.3% male. Physicians: 33.1% male | Patients: Median age: 49.5. Physicians: not given | Patients: 51.3% white. Physicians: not given | Clinician-focussed teachable moment communication process. | AAC training - Ask, Advise, Connect | Average 14.5 months |
| Funk 2005 | RT | Australia, Belgium, Denmark, England, New Zealand, and Spain | Alcohol | 464 GPs | GPs were included if they were registered practitioners who delivered general medical services | GPs were excluded if they moved away from the study area, provided only locum or emergency medical services, they were on extended leave, they had been involved in pilot work for this study, or if a practice partner had already been approached for the study. | 76% male | 45 years | not given | One face to face training session related to brief intervention programme | Written instructions | 3 months |
| Girvalaki 2018 | cNRT | Crete | Smoking | 24 GPs | Eligible patients were 18 years or older; current smokers; seen in practice for a nonurgent medical visit or prescription and able to read/ understand Greek | Not described | Smoker’s sex: 57.6% male | Smoker’s age: 48 years | Smokers: 97.9% Greek | Training, practice, and patient tools | Usual care | 4 months |
| Haller 2014 | cRT | Switzerland | Alcohol | 33 family physicians (1 withdrew after randomisation before patient recruitment) | Young person (15-25) consulting at the practice | Acute illness; severe mental disturbance; substance abuse requiring immediate attention; previous treatment for dependence; inability to read or understand French; any other disorder impacting their ability to consent | Physicians: 58% male. Patients: 46.8% male | Physicians: median age 50.5 years. Patients: 18.5 years | Not described | 2 x training sessions (3h and 2h) simulations, practicing BIs, feedback | Usual care | 12 months |
| Haskard 2008 | RT | West Coast, USA | Multiple | 80 physicians | Patients had to be literate and able to communicate in English or Spanish. Spanish speaking patients could be included if their physician was fluent in Spanish and both the interaction and patient questionnaire were completed in Spanish | No physicians who volunteered were excluded | 63% male | Physicians: 37.3 years (SD = 10.1) | not given | 18 hours of training over 3 months with 90-120 minutes of coaching (review of consultations) alongside | No training (received after trial completed) | 6 months |
| Hilbink 2012 | cRT | Netherlands | Alcohol | 82 practices with 124 GPs | All GPs in the practice had to agree to participate | Not described | Patients: 69.7% male | Patients: 47.5 years | Not given | Educational visits by a trained facilitator, personal feedback | Mailed guidance | 2 years |
| Houston 2013 | cRT | Dental practices from Alabama, Georgia, Florida and North Carolina, USA | Smoking | 143 practices |  |  | Patients: 47.8% male | Patient: median age = 37 | Patients: 84.7% white | OralCancerPrevention.org - educational cases, patient education, forum, email reminders | Usual care | 6 months |
| Hudmon 2018 | cRT | Connecticut and Washington state, USA | Smoking | 64 pharmacies | Listed pharmacy in either state | Not described | Not given | Not given | Not given | Academic detailing (on-site training) | Printed materials | 12 months |
| Joseph 2004 | cRT | Veterans Affairs Medical Centers, Minnesota, USA | Smoking | Not given | Existence of a referral-based smoking cessation program that treated a minimum of 50 patients a year, Information Resource Management capacity for data collection, Institutional Review Board capacity and evidence of commitment to the project | Small sites, lack of experience with research. Facilities serving predominantly psychiatric patients | Patients: 96% male | Patients: 64 years | Patients: 75% white | 2-day training meeting including smoking as a vital sign approach, delivery of brief intervention to all smokers, use of pharmacological therapy and further site visits | Usual care | 12 months |
| Jumbe 2022 | cRT | 60 community pharmacies in England and Wales | Smoking | 60 pharmacies; 30 intervention, 30 control | Provided stop smoking services and were identified from lists provided by NHS service commissioners in each recruiting area. Patients: all current smokers 18 or older who joined the stop smoking services in participating pharmacies and who attended their first session | None specifically given | 50.16% Male | Mean age smokers 45.83 (SD 14.31) | not given | STOP training intervention, 1 x half-day 3hr session for pharmacists | Usual care | 6 months |
| Kaner 2003 | cRT | Seven health districts across northern England | Alcohol | 144 practices | At least one nurse who would not be away from the practice for more than 2 weeks | Not described | Nurses 99% female | Nurses: 45 years (SD 8) | Not given | 34-minute outreach visit detailing brief intervention procedures and pre-empting problems | Letter covering guidelines | 3 months |
| Keller 2000 | cRT | Hesse and Theringden, Germany | Multiple | 74 primary care physicians | Geographical criteria (for physicians), patients had newly diagnosed or previously untreated cardiovascular risk factors | Patients were excluded if cardiovascular disease or other serious diseases were known or if they had received a cardiovascular risk factor intervention in the past | Patients: 57% male | Patients: 49 years | Not given | One day interactive workshop focussing on the trans-theoretical model and general counselling strategies for behaviour modification | Usual care | 12 months |
| Kottke 1989 | cRT | Minnesota family practice, USA | Smoking | 44 physicians | Not described | Not described | GP: 84.9% men; patients 33% men | GPs: 40 years; patients 40 years | not given | 6-hour training covering dangers of smoking, intervention skills, counselling skills and planning the practice for smoking cessation | Usual care | 12 months |
| Lennox 1998 | cRT | Aberdeen city, UK | Smoking | 16 practices | All practices (other than practices the research staff worked at) invited to participate | Staff member had attended pilot workshop prior to joining practice, impending large staff changes, staff member worked for more than one practice | Not given | Not given | Not given | Interactive approach to smoking cessation, some didactic teaching on the Stages of Change model | Usual care | 14 months |
| Malta 2016 | cNRT | Sao Paulo State, Brazil | Multiple | 43 doctors and nurses | All doctors providing low risk antenatal care within the public health network of Botucatu municipality | Disaffiliation with municipal primary care staff | Not given | Dr and nurses: 36.6 years | Not given | 16h intervention package including an introductory course and 3 workshops | Usual care | 12 months |
| McRobbie 2008 | cRT | Eat London, UK | Smoking | 91 GPs | Not given | Not given | Not given | Not given | Not given | Training session including practical guidelines and simple skills to refer smokers for treatment | Referral guidance by post | 3 months |
| Mejia 2016 | cRT | Argentina | Smoking | 254 physicians; 1378 smokers | Physicians who saw more than 100 patients a month in primary care specialties within the 6 health systems 3 months before the study began | Not described | Physicians: 47% men; patients:19% men | Physicians: 44.5 years; patients median age 35-49 | not given | 2 x 3-hour didactic sessions | Usual Care | 12 months |
| Moore 2003 | cRT | Northern and Yorkshire region of England | Obesity | 44 practices | All general practitioners and practice nurses in the 44 GP practices were eligible to participate | Not described | 26% male | 48 (11) years | not given | 3 x 90-minute training sessions about feasible interventions and how to deliver them | Usual care | 12 months |
| Olano-Espinosa 2013 | cRT | Spain | Smoking | 35 primary health care centres, 830 health professionals, 5970 smokers | All nurses and doctors from the 35 clinics were included. Patients were at least 18 years old, identified as smokers, attended a consultation during the follow up period | No exclusion criteria for practices, Patients were excluded if they were immobile, terminal, in geriatric centres or "absent" (someone else had come in their place). | Healthcare professionals: 50% male; patients: 47% male | Healthcare professionals: 45; Patients: 44 years | Not given | 6-hour training (4 x 90min) - training based on stage of change model, clinical cases and role playing | Usual care | 12 months |
| Ornstein 2013 | cRT | 15 states of USA | Alcohol | 20 primary care practices | First 20 practices volunteering for the study | None described | 44% male | Patients: 60 years (providers not given) | Not given | Network meeting; 2 on-site educational visits; all received passive dissemination of guidelines, electronic health record update, audit, and feedback | all practices received passive dissemination of guidelines, Electronic Health Record update, audit, and feedback | 12 months |
| Prokhorov 2010 | cRT | Texas, USA | Smoking | 16 communities of 87 physicians and 83 pharmacists | At least 18 years, able to speak and write in English or Spanish | Not described | Physicians 57% male; pharmacists 47% male; patients 30% male | Physicians: 44 (11) years; pharmacists 44(13) years; patients 45 (15) years | Physicians 69% white; pharmacists 67% white; patients 81% white | Smoking cessation continuing education training | Skin cancer training | 12 months |
| Ribeiro 2011 | cRT | District of Lisbon, Portugal | Alcohol | 73 doctors | Random sample of doctors | Not described | 39.7% male | 53.5 years | Not given | Training in identification and counselling of patients with risky alcohol consumption | Usual care | 9 months |
| Rosario 2022 | cRT | Portugal | Alcohol | 12 clinics | All primary health care units will be eligible to participate | PHC units will be excluded if they have less than 55 patient lists, or if they have a specific alcohol programme implemented in their practice | Physicians: 10% male | Physicians: 43 years | Not given | 4 sessions covering evidence of alcohol harm, how to screen and deliver brief interventions, share experiences, personalised plans, and practice of brief interventions | Usual care | 12 months |
| Ruf 2010 | cRT | South Baden and South Württemberg, Germany | Alcohol | 69 practices | Use of broadband internet, at least one practice nurse | Non defined by study | GPs: 65.2% male; patients: 73.6% male | GPs: 51.5 years; patients: 54.2 years | Not given | Online and training programme for general practitioners. Additionally, education for the whole practice | Access to the system with no training | 3.5 months |
| Sinclair 1998 | cRT | Grampian, Scotland | Smoking | 62 pharmacies | All pharmacies in Grampian area | City pharmacies due to a concurrent training initiative | Patient: 38% men | Patient: 41 years | Not given | Training package based on the stage of change model of smoking cessation | Usual care | 9 months |
| VanLieshout 2016 | cRT | Dutch general practice | Multiple | 34 general practices | Patients had to be adults (aged 18 or older), have a high risk of CVD, no established CVD, capable of providing informed consent | Diabetes mellitus, pregnancy and lactation, terminal illness, cognitive impairment, and poor language skills | PNs: Not given; Patients: 65% male | Practice nurse: 42 years; Patients: 72 years | Not given | Structured feedback; access to online education programme; and a flowchart for dealing with depressive symptoms | Usual care | 6 months |
| Verbiest 2014 | cRT | The Netherlands | Smoking | 49 GPs, 3401 patients (677 smokers) | No more than 5 self-reported stop smoking advice per week | No participating colleague in the same practice | 38.6% male | 52.5 years | not given | 1 hour training for GPs on the 5A behavioural change model and a toolkit | Usual care | 9 months |
| Welzel 2021 | cRT | Central Germany | Obesity | 50 practices | None for GPs; patients BMI >30kg/m2 age 18-60, not acute illness presentation | Patients do not meet inclusion criteria | Patients 37.8% male; GPs 38.8% male | GPs: 48.6 years; patients 43.3 years (SD 10.7) | not given | Online tutorial access | Usual care | 12 months |
| **Electronic Patient Record** | | | | | | | | | | | | |
| Fiore 2019 A | cRT | Wisconsin, USA | Smoking | 12 clinics in health system A (unclear) | Clinics had to provide primary care, have a volume of >60 patients a week, >= 3 primary care clinicians, have received prior training in F2Q to the Wisconsin Tobacco Quitline and willingness to accept random assignment. | Not described | Patients: 43.7% male | Patient:55.4 years | Patients: 97.3% white | eReferral process to Quitline on electronic health record (with training on how to. Use this) | Faxed referral | 6 months |
| Fiore 2019 B | cRT | As above | As above | As above | As above | As above | Patients: 41.8% male | Patient: 55.0 years | Patients: 76.4% white | As above | As above | 6 months |
| Houston 2015 | cRT | US | Smoking | 174 practices | All smokers in the practices were eligible for referral | Not described | Not given (trial 1) | Not given (trial 1) | Not given (trial 1) | ePortal with online referrals to Web-Assisted Tobacco intervention | Paper referrals | 6 months |
| Rindal 2022 | cRT | Midwest, USA | Smoking | 15 non-academic primary care dental clinics | For a dental practitioner to be eligible for the study, they could be either a dentist or a dental hygienist. They had to use an EagleSoft or Dentrix electronic dental record (EDR), provide estimates of their patient attributes, agree to participate in training along with their staff, and agree to be randomized to one of the study arms. Patients had to be 18 or over, smoked cigarettes and was not yet exposed to clinical decision support | Dental practitioners whose practices were limited to specialty services other than periodontal care or who practiced at an educational or government institution or in a Federally Qualified Health Centre (FQHC) were not eligible to participate. | 60% male | 42.3 (15.8) | 42% white | The CDS tool required the dental provider to assess 3 health behaviour variables for all patients who reported smoking cigarettes | Usual care | 6 months |
| Minian 2022 | RT | Ontario, Canada | Multiple | 5331 patients | Treatment-seeking smokers enrolled at the STOP program at one of the partnering primary care settings, reported physical activity and fruit and vegetable consumption that was lower than national guidelines. English--speaking and have provided at least piece of contact information (for follow up) | Not meeting inclusion criteria | 48% male | 53 (SD 13) years | not given | Diet and physical activity clinical decision support tool | Usual care | 6 months |
| Boston 2023 | cRT | Community health Centres in USA (low-income patients) | Smoking | 70 community health centres | Included patients were medication-naive and aged 40 to 75 years with ≥1 uncontrolled cardiovascular disease risk factor, with known diabetes or cardiovascular disease, or ≥10% 10-year reversible CVD risk. | Not meeting inclusion criteria or declined to participate | Patients: 47.8% men | 58.5 years (8.8) | Patients 65.7% white | Clinical decision support prompt and implementation resources | Usual Care | 18 months |
| **Facilitated relay of information** | | | | | | | | | | | | |
| Grant 2014 | cNRT | Kaiser Permanente Northern California | Physical Activity | 11 medical centres; 696,267 adults | Adults aged 18-89 years, continuous membership plan, visited their PCP during the study observation period | Switched medical affiliation during the study period, recent hospitalisations or pregnancies, presence of comorbidities that might limit exercise capacity | Patients: 47.7% | Patients: 51.4 years | Patients: 46.6% white | Change to medical assistant workflow and an added feature to the electronic medical record | Did not have exercise as vital sign (EVS) program | 12.5 months (average time post implementation for 4 clinics) |
| Wadland 2007 | cRT | Michigan, USA | Smoking | 87 practices; 308 clinicians | Practices offered usual primary care, agreed to participate, completed baseline survey forms | Not described | Physicians: 53% male | Patients: 23% >65 | Patients: 80% white | Comparative feedback report to clinicians | Usual care | 18 months |
| Unrod 2007 | cRT | Boroughs of New York, USA | Smoking | 70 physicians | Physicians: internal or family medicine specialty, plans to continue practicing in current location for at least 1 year, at least 75 patient visits/week, primarily English-speaking patients, fewer than 25% geriatric patients. Patients: At least 18 years, smoked in the past 7 days, smoked more than 100 cigarettes in their lifetime, English-speaking, to keep their physician for the next 1 year. | Not meeting inclusion criteria | Patients: 39% male | Patients: 43 years | Patients: 62% white | Patient smoking report collected by research staff for review by physician | Usual care | 6 months |
| Bentz 2007 | cRT | Providence Health Systems, an integrated delivery system in Portland, Oregan. | Smoking | 19 clinics. Control (n=9); Intervention (n=10) | Both fax and self-referrals included, patients gave consent, had to be smokers | Not specified | Patient: 33.7% male; Provider: 50.5% male | Patient: 52.5 | Not given | Clinicians received feedback on Asked, Advised, Assessed and Assisted components of smoking cessation | No feedback | 12 months |
| Saitz 2003 | cRT | Boston University Medical Centre, Boston, MA, USA | Alcohol | 50 physicians | Faculty and resident PCPs in an urban academic medical practice | Physicians who saw <80 patients in previous 3 years or who anticipated leaving the practice within 6m | Patient: 63.5% male; Physicians: 55% male | Patient: 43 years; Physicians: 34.5 years | Patient: 19% white Physicians: 66% white | Sheet of paper attached to notes prior to physician visit with the results of the alcohol intake, assessment of alcohol problems, advice for next steps (referral, discussion etc) | No relay of information to physicians | 6 months |
| **Financial Incentives** | | | | | | | | | | | | |
| Bardach 2013 | cRT | New York City, USA | Smoking | 84 practices | Practices with <10 clinicians, having at least 200 eligible patients for measurement, at least 10% Medicaid or uninsured patients, have been using the electronic health record for at least 3 months | Not given | Patients: 41% male | Patients: 46 years | Not given | Financial incentives and bench lined quarterly reports | Benchmarked quarterly performance | 1 year |
| Coleman 2007 | ITS | English primary care | Smoking | 1,607,782 patients in 2004 | Patients aged 15-75 years | Not described | Not given | Not given | Not given | Introduction of new contract with financial incentive (QoF) for smoking brief intervention in 2004 | N/A | 12 months |
| Fichera 2016 | ITS | English primary care | Multiple | 11,270 in BMI sample (used in extraction) | Individuals reporting at least one condition incentivised by the QOF | Not given | Patients: 47% male | Patients: 55 years | Not given | Introduction of QOF for obesity, smoking, alcohol (BMI chosen as first outcome) | N/A | 5 years post QOF introduction 2004 - 2009 |
| O'Donnell 2020 | ITS | England | Alcohol | 4,278,723 | Individual practices were eligible if their data were published after the Acceptable Mortality Reporting and Acceptable Computer Usage dates. Patients were eligible if they were newly registered (defined as within the previous 12 months), aged 16-99, and registered with a practice that had been contributing to THIN for at least 2 years prior to the patients' registration date | Patients with recorded ages of 100+ | Patients: 57.5% male | Patients: 38 years | Not given | Introduction of financial incentives for alcohol screening | N/A | 11 years |
| Rieckmann 2018 | ITS | Oregan, USA | Multiple | 516,708 participants total | Individuals aged 18-64 | Individuals dually enrolled in Medicaid and Medicare, those eligible for Medicaid under Medicaid expansion, people not aligned to a CCO | Study population: 30% male | Study population: 35 years | Study population: 70% white | Incentive payments to CCOs when they implement SBI. | N/A | 30 months follow up time |
| Roski 2003 | cRT | Midwest, USA | Smoking | 37 clinics | For patient tobacco endpoints - 18 or older, having a telephone number in their records, having an office visit 90 days prior to July 10, 2000 | Not described | Not given | Not given | Not given | Financial incentive for meeting smoker id and advice target (and target assessment through chart abstraction) | Guideline dissemination | 6 months |
| Szatkowski 2016 | ITS | England | Smoking | 3,337,881 patients in the database per month | All patients aged >16 years who contributed to the database from April 2004 to March 2013 | Not given | Not given | Not given | Not given | QOF smoking revision | N/A | 1 year |
| **Multicomponent interventions** | | | | | | | | | | | | |
| Ackermann 2005 | cRT | Seattle, USA | Physical Activity | 31 physicians and nurse practitioners; 336 patients | All staff and fellow physicians, nurse practitioners, and physician assistants caring for primary care patients were eligible. Patients were eligible with written consent, aged 50 or older, lived within 25 miles of the VA. | Practitioners on a leave of absence. Patients with severe cognitive deficits. | Patients: 99% male | Patient age: 66 years. Physician age: 43 | Not described | 10-minute individualised training session and prompts for each patient detailing their stage of change | Analogous tobacco training | 4 months |
| Anderson 2016 | cRT | Catalonia, England, the Netherlands, Poland, and Sweden | Alcohol | 120 primary care health units (24 in each country) | Any fully trained full- or part- time medical practitioner, nurse, or primary care assistant with a permanent contract | None given | Patients: 55% male; providers: 26% male | Patient: 53 years; Provider 47 years | Not given | Training, financial reimbursement, referring identified heavy drinking patients to an internet-based method of delivering advice. | Printed guidance | 12 weeks |
| Asadi-Aliabadi 2023 | cRT | 4 Iranian districts | Multiple | 31 community health centres | At least 2 healthcare providers, rural - without a satellite village | Excluded fields with specific characteristics that differentiate from other fields in the district | 50% men | 49 years | not given | Goal setting, education, operational planning, and incentive payments | Usual care | 12 months |
| Bailey 2023 | cNRT | USA | Smoking | 8 community health centres | The study included adults (aged ≥18 years) with ≥1 primary care visit between August 2016 and September 2019 to ≥1 of the 8 community-based primary care study clinics that primarily serve uninsured or publicly insured patients. | Not meeting inclusion criteria | 30.4% male | Median 35-64 | 49.6% white | EHR referral functionality and expansion of staff roles | Usual care | 24 months |
| Balasubramanian 2022 | ITS | USA | Smoking | 1795 practices | Smaller practices (< 10 FTE clinicians), had to have an electronic health record | Not meeting inclusion criteria, declined to participate, other reasons | not given | Not given | Intervention 59.8% white | ITS | ITS | 2 years |
| Goodfellow 2016 | cRT | East Midlands England | Obesity | 28 general practices | All practices in the East Midlands of England (except Derbyshire Clinical Commissioning Group) | The exclusion criteria for practices were (a) participation in another study of obesity and assessing similar outcomes during the previous year and (b) if the practice had recently changed or were planning to change their computer system over the trial period | Patient: 52% male | Patient: 51.3 years | Patient: 68.6% white | Training, team discussion, discussion about sample questions, booklet for patients, guidelines summary | Usual care | 9 months |
| Harris 2017 | cRT | Australian states | Multiple | 32 practices | Eligible patients 40-69 years without CVD, renal disease or diabetes who had visited the practice within 1 year; proficient in English; had cognitive awareness | severe mental illness, substance abuse, pregnancy | Patients: 30.4% male | Patients: 55 years | Not given | Clinician education; audit and feedback; practice facilitation visits | Usual care N/A Usual care | 12 months |
| Kowitt 2022 | cRT | North Carolina, USA | Smoking | 28 practices | 10 or fewer clinicians and had an HER | Not described in this paper | Patients:40.1% male | Not given | Patients: 73.1% white | Healthy Heart Now (HHN): online coaching and support, HER practice population management dashboard, educational tools, Coach worked with practices on Plan-Do-Study-Act cycles | Usual care | 6 months |
| Lee 2023 | cRT | Washington State, USA | Alcohol | 22 practices (19 sites, 3 practices on same site) | All patients who were at least 18 years old with a visit(s) to trial primary care practice(s) between January 1, 2015, and July 31, 2018. | Not described | 42% male | 48 (18) years | 70% white | EHR clinical decision support, performance feedback, practice facilitation | Usual care | 2 years |
| Little 2009 | cRT | USA | Smoking | 14 dental practices | Not described | Excluded smokers where they had only been seen by a dentist at a new patient exam | 42.6% male | 46 years | Approximately 84% whit *demographic data is from post-visit sample of patients completing questionnaire | Training to offer brief tobacco counselling and encourage patients to talk by telephone with a trained tobacco counsellor, performance monitoring and feedback, facilitated relay of information | Usual care | 14 months |
| McElwaine 2014 | cNRT | New South Wales Australia | Multiple | 17 community health facilities; 571 nurses and allied healthcare professionals | Adults, at least 1 face to face appointment in the past 2 weeks, not previously selected, spoke English, mentally and physically capable of completing the interview, not involved in another community healthcare study | Not described | Participants: 35% male | Median age of participants: >=60 | Not given | Electronic medical record modification; monthly performance reports; practice change support officers; email helpline; clinician report pack | Usual preventive care | 12 months |
| Patwardan 2012 | RT | South -Central Wisconsin, USA | Smoking | 16 pharmacies; 30 pharmacists | 1) they belonged to a specific large national chain pharmacy, 2) they were located within 75 miles radius from Madison, and 3) they were in an area of lower socioeconomic status than the state average | Not specified | 50.5% male | 39.6 years | not given | Training, workflow recommendations, poster, support visit (+ control) | Cards and informal presentation | Unclear - 5m (July 2008 pharmacy recruitment, data gathered November 2008) |
| Rose 2008 | cRT | 21 primary care practices across the USA | Alcohol | 21 primary care practices | Practices members of the Practice Partner Research Network (PPRNet) First 22 practices to volunteer | Not described | Patient: 43% male (intervention arm) | Patient: 62 years (intervention arm, do not give control, but say statistically similar) | Not given | Academic detailing, performance reports, network meetings | Received performance feedback | 2 years |
| Sturgiss 2023 | ITS | ITS | Alcohol | 17,107 (plus clinic 6 regular patients - not given) | ITS | ITS | not given | Not given | not given | ITS | ITS | 10 months |
| Twardella 2007 | cRT | Germany | Smoking | 41 medical practices; 45 general practitioners | Smoked at least 10 cigarettes per day; aged 36-75 years | Not described | patients: 49.5% male | Patients: Median 45-54 years | not given | Physician group training and direct physician payments for every smoker not smoking at 12 months | Usual care | 12 months |
| van Beurden 2012 | cRT | Netherlands | Alcohol | 77 general practices | Practices could only enrol if all GPs within the practice agreed to participate | Refused to participate | GPs 33% male | GPs: 47.4 years | not given | Educational training session, reminder card, feedback report, facilitation of the cooperation with local addiction services, outreach by trained facilitator | Usual care | 12 months |
| Wiggers 2017 | cRT | New South Wales, Australia | Multiple | 56 practices | Adult health centres that provided: community nursing, diabetes, aged care, counselling, dietetics, psychology, physiotherapy, and occupational therapy services | Child based services, in-patient services, specialist community services | Client: 41% male | Client: median age 60+ | Not given | 6 key strategies: leadership and consensus processes, enabling systems, educational meetings and training, audit and feedback, practice change support, and practice change information and resources. | Usual care | Average 24 months |
| Young 2002 | cRT | Sydney, Australia | Smoking | 60 family practitioners from 39 practices | All family practitioners in a defined geographical area around Sydney | Worked fewer than 2 days a week, planning to leave within 6 months, on extended or maternity leave, did not employ a receptionist, already participating in a clinical audit, more than 50% of their patients spoke a language other than English at home. Patients were ineligible if they did not speak or read English, were unable to read or understand the questionnaire, were too sick or distressed to participate, or had participated previously. | patients 39% male; physicians 38% male | Patients median 45 years; physicians 48 years | Not given | Audit and feedback, resources and skill training, prompt sheet, reminder for medical records, patient brochures | Cervical cancer training | Max 6 months |
| **Continuous Quality Improvement** | | | | | | | | | | | | |
| Yano 2008 | cRT | Veterans Health Administration health care networks in SW USA | Smoking | 18 practices, 925 smokers’ intervention; 1016 control practices | 3 or more primary care providers and 3000 or more primary care patients | research team's home institution, practice had insufficient time to participate/ patients excluded if not eligible, refused, phone problems or not located | Patients: 93% male | Patients: 57 years | Patients: 64% white | QI strategies | Usual care | 12 months |
| **Team Changes** | | | | | | | | | | | | |
| Flocke 2020 | CBA | Ohio, USA | Smoking | 8 clusters, 81 MA/RNs | Eligibility for QL programme included 18 or older, Medicaid insurance, no insurance or pregnant. All patients seen by MA/RNs during the observation period were eligible for tobacco assessment | Not described | Patients: 30.9% male | Patients: Median age: 49.5. Physicians: not given | Patients: 48% white | New HER section to Ask-Advice-Connect to be implemented before clinic appointments by medical assistants or registered nurses as part of vital signs. | Usual care | 12 months |
| Alageel 2019 | ITS | England | Multiple | 450,801 | Health check cohort included participants in England aged 40-74 years who had a health check between 1/4/23and 31/12/2013 | A diagnosis of ischaemic heart disease, stroke, or diabetes, or were treated with antihypertensive drugs or statins before the date of the health check. | 51.9% male | Patient median = 50 | Not given | Introduction of the NHS health check for the primary prevention of cardiovascular disease and related disorders | Usual care | 6 years |
| Schwartz 2014 | cRT | New York Harbor, USA | Smoking | 20 teams (13 in the arms analysed) | All PACT teams | If there were no primary care physicians | 94% males | 63 years | not given | Addition of a Panel Management Assistant who identified gaps in care, conducted patient outreach, supported hypertension and smoking outcomes | Usual care | 8 months |
