# Supplementary material for: Strategies to improve the implementation of preventive care in primary care: a systematic review and meta-analysis
Source: BMC Med. 2024 Sep 27;22:412. doi: 10.1186/s12916-024-03588-5 (PMC11437661; doi:10.1186/s12916-024-03588-5)
Supplement: Supplementary file 8 — Additional file 8: Table S1 - Prediction interval calculation and interpretation. [file 12916_2024_3588_MOESM8_ESM.docx]

| Intervention category | Odds ratio (OR) | 95% Confidence interval (CI) | Interpretation of CI | 95% Prediction interval (PI) | Interpretation of PI |
| --- | --- | --- | --- | --- | --- |
| *Results for process outcomes* |  |  |  |  |  |
| Clinician reminders | 3.46 | 1.72 to 6.96 | We are 95% certain the true average OR is between 1.72 and 6.96 | 0.30 to 39.6 | Given 7 studies with this heterogeneity,  it is plausible that settings or populations exist in which the OR is as low as 0.30 or as high as 39.6 |
| Clinician education | 1.86 | 1.46 to 2.46 | We are 95% certain the true average OR is between 1.50 and 2.59 | 0.58 to 6.20 | Given 29 studies with this heterogeneity, it is plausible that settings or populations exist in which the OR is as low as 0.58 or as high as 6.20 |
| Electronic patient record | 2.00 | 0.89 to 4.48 | We are 95% certain the true average OR is between 0.89 and 4.48 | 0.12 to 32.5 | Given 5 studies with this heterogeneity,  it is plausible that settings or populations exist in which the OR is as low as 0.12 or as high as 32.5 |
| Facilitated relay of information | 1.95 | 1.10 to 3.46 | We are 95% certain the true average OR is between 1.10 and 3.46 | 0.15 to 27.1 | Given 4 studies with this heterogeneity,  it is plausible that settings or populations exist in which the OR is as low as 0.15 or as high as 27.1 |
| Financial incentives | 1.10 | 0.82 to 1.48 | We are 95% certain the true average OR is between 0.82 and 1.48 | N/A | Given 2 studies it is not possible to estimate a prediction interval. |
| Multicomponent | 3.10 | 1.60 to 5.99 | We are 95% certain the true average OR is between 1.60 and 5.99 | 0.27 to 35.2 | Given 12 studies with this heterogeneity,  it is plausible that settings or populations exist in which the OR is as low as 0.27 or as high as 35.2 |
| Team changes | 9.80 | 0.16 to 584.2 | We are 95% certain the true average OR is between 0.16 and 584.2 | N/A | Given 2 studies it is not possible to estimate a prediction interval. |
| *Results for behavioural outcomes* |  |  |  |  |  |
| Clinician reminders | 1.06 | 0.91 to 1.23 | We are 95% certain the true average OR is between 0.91 and 1.23 | 0.40 to 2.82 | Given 3 studies with this heterogeneity, it is plausible that settings or populations exist in which the OR is as low as 0.40 or as high as 2.82 |
| Clinician education | 1.03 | 0.91 to 1.15 | We are 95% certain the true average OR is between 0.91 and 1.15 | 0.85 to 1.24 | Given 15 studies with this heterogeneity, it is plausible that settings or populations exist in which the OR is as low as 0.85 or as high as 1.24 |
| Electronic patient record | 0.92 | 0.53 to 1.58 | We are 95% certain the true average OR is between 0.53 and 1.58 | N/A | Given 2 studies it is not possible to estimate a prediction interval. |
| Facilitated relay of information | 1.10 | 0.78 to 1.56 | We are 95% certain the true average OR is between 0.78 and 1.56 | 0.03 to 37.4 | Given 3 studies with this heterogeneity, it is plausible that settings or populations exist in which the OR is as low as 0.03 or as high as 37.4 |
| Multicomponent | 1.19 | 0.89 to 1.58 | We are 95% certain the true average OR is between 0.89 and 1.58 | 0.39 to 3.65 | Given 4 studies with this heterogeneity, it is plausible that settings or populations exist in which the OR is as low as 0.39 or as high as 3.65 |
